# Evolution of the Gut Microbiome in HIV-Exposed Uninfected and Unexposed Infants during the First Year of Life

**DOI:** 10.1128/mbio.01229-22

**Published:** 2022-09-08

**Authors:** Conner L. Jackson, Daniel N. Frank, Charles E. Robertson, Diana Ir, Jennifer M. Kofonow, Mahlodi P. Montlha, Eleonora A. M. L. Mutsaerts, Marta C. Nunes, Shabir A. Madhi, Debashis Ghosh, Adriana Weinberg

**Affiliations:** a Department of Biostatistics and Informatics, Colorado School of Public Health, University of Colorado Anschutz Medical Campus, Aurora, Colorado, USA; b Department of Medicine, University of Colorado Anschutz Medical Campus, Aurora, Colorado, USA; c South African Medical Research Council Vaccines and Infectious Diseases Analytics Research Unit and Department of Science and Technology/National Research Foundation South African Research Chair Initiative in Vaccine Preventable Diseases, Faculty of Health Sciences, University of the Witwatersrand, Johannesburg, South Africa; d African Leadership in Vaccinology Expertise, Faculty of Health Sciences, University of the Witwatersrand, Johannesburg, South Africa; e Department of Pediatrics, University of Colorado Anschutz Medical Campus, Aurora, Colorado, USA; f Department of Pathology, University of Colorado Anschutz Medical Campus, Aurora, Colorado, USA; University of Kentucky

**Keywords:** HIV, HIV-exposed uninfected infants, gut microbiome, breast milk microbiome, pregnant women with HIV, human immunodeficiency virus

## Abstract

HIV-exposed uninfected infants (HEU) have abnormal immunologic functions and increased infectious morbidity in the first 6 months of life, which gradually decreases thereafter. The mechanisms underlying HEU immune dysfunctions are unknown. We hypothesized that unique characteristics of the HEU gut microbiota associated with maternal HIV status may underlie the HEU immunologic dysfunctions. We characterized the infant gut, maternal gut, and breast milk microbiomes of mother-infant pairs, including 123 with HEU and 117 with HIV-uninfected infants (HUU), from South Africa. Pan-bacterial 16S rRNA gene sequencing was performed on (i) infant stool at 6, 28, and 62 weeks; (ii) maternal stool at delivery and 62 weeks; and (iii) breast milk at 6 weeks. Infant gut alpha and beta diversities were similar between groups. Microbial composition significantly differed, including 12 genera, 5 families and 1 phylum at 6 weeks; 12 genera and 2 families at 28 weeks; and 2 genera and 2 families at 62 weeks of life. Maternal gut microbiomes significantly differed in beta diversity and microbial composition, and breast milk microbiomes differed in microbial composition only. Infant gut microbiotas extensively overlapped with maternal gut and minimally with breast milk microbiotas. Nevertheless, exclusively breastfed HEU and HUU had less divergent microbiomes than nonexclusively breastfed infants. Feeding pattern and maternal gut microbiome imprint the HEU gut microbiome. Compared to HUU, the HEU gut microbiome prominently differs in early infancy, including increased abundance of taxa previously observed to be present in excess in adults with HIV. The HEU and HUU gut microbiome compositions converge over time, mirroring the kinetics of HEU infectious morbidity risk.

## INTRODUCTION

*In utero*-HIV-exposed uninfected infants (HEU) have a higher incidence of hospitalization and 2- to 4-fold higher mortality rate than HIV-unexposed uninfected infants (HUU) in low-and-middle-income countries (1–15). In the United States, Canada, and other areas with improved health care, the risk of death is similar in HEU and HUU, but recent studies have reported more frequent hospitalizations due to infections in HEU (16–20). The infections with high morbidity and mortality in HEU are caused by common childhood pathogens, including respiratory syncytial virus (RSV) and Streptococcus pneumoniae (14, 21–25). Insufficient passive protection due to low transplacental transport of maternal antibodies in women with HIV might account for the increased morbidity of infections in HEU during early infancy (22, 26). However, we showed that concentrations at birth of antibodies against respiratory pathogens, including RSV and S. pneumoniae, were similar in HEU who developed medically attended lower respiratory tract infections in the first 6 months of life and those who did not, suggesting that the immune responses of HEU to infections are insufficient to control these pathogens (27). Multiple phenotypic and functional immunologic characteristics differentiate HEU and HUU in the first year of life, particularly in cell-mediated immunity (19, 20, 28–30). These dysfunctions may explain the increased vulnerability of HEU infants to infections. However, the mechanisms underlying immunologic dysfunctions in HEU are not currently understood.

The gut microbiome has an important role in the education of the infant immune system. The human gut harbors 12% to 20% of the total lymphocytes, and most importantly, it is a site of innate and adaptive T cell maturation second only to the thymus (31, 32). Myeloid and lymphoid immune cells acquire specific characteristics through transcriptional and epigenetic reprogramming and modulation of transcription factor binding directed by the gut microbiota (33–35). Gut-imprinted myeloid and lymphoid cells exit the gut to populate intestinal lymph nodes, the peripheral blood, and other tissues (36–41). The composition of the infant gut microbiome undergoes sequential changes after birth, influenced primarily by the delivery mode, maternal microbiome, and maternal and infant diet (42–50). Other factors, including gestational age at birth, antibiotic therapy, genetic background of the host, and geographic location, also contribute to the composition and structure of the microbiome (33, 49–58). Although subject to many changes in the first 1 to 2 years of life, the infant gut microbiome evolves in a scripted fashion until it acquires adult characteristics (59, 60).

Pioneering work by Bender et al. showed differences in gut microbial alpha diversity of 1- to 4-month-old HEU and HUU examined cross-sectionally at a single time point (61). The authors did not find sufficient differences in either breast milk or skin microbiotas of mothers with and without HIV to explain HEU gut dysbiosis and did not analyze the maternal gut microbiome. Subsequently, D’Souza et al. compared the structure of the gut microbiome at 6, 16, and 24 weeks of age in HEU receiving co-trimoxazole prophylaxis or not and showed that co-trimoxazole administration may also contribute to the dysbiosis observed in HEU (62). Two other cross-sectional studies that compared HEU and HUU gut microbiomes at approximately 2 years of life found similar alpha or beta diversities in the two groups (63, 64).

Breast milk, oral, vaginal, and maternal gut microbiotas contribute to the formation of the infant microbiome in the beginning of life, with the gut microbiota making the largest contribution (65). In addition, strictly anaerobic bacteria are shared by the breast milk and maternal gut, which led to the proposal of a gut-breast axis whereby dendritic cells transport bacteria from the gut to the mammary gland (66, 67). Due to pH and other ecologic requirements, the maternal vaginal microbiota may only transiently colonize the infant gut (65). Previous studies have shown differences in the composition of the gut microbiotas of people with and without HIV (68, 69). After early controversy whether the differences originated from HIV infection or sexual practices of males who have sex with males, it was established that HIV infection by itself was associated with specific microbiome characteristics, including in pregnant and nonpregnant women (68). These conclusions were consistent with the bidirectional relationship between microbiota and host immune system, whereby the host immune responses to commensals play an important role in shaping the microbiome (52).

The overarching hypothesis of this study is that gut dysbiosis in HEU contributes to immunologic dysfunctions in early life. Moreover, the composition of the gut microbiome can be modified by adjusting the diet and other determining factors. Here, we report on the evolution of the gut microbiome in HEU in comparison with that in HUU during the first 62 weeks of life and the relationship of the maternal gut, breast milk, and infant gut microbiotas in a longitudinal cohort of 240 mother-infant dyads.

## RESULTS

### Characteristics of the study population.

This study enrolled 240 mother-infant pairs, including 123 mothers with HIV and 117 without HIV, between June and December 2017. Notable differences between the mothers with and without HIV were higher chronological age and parity and lower body mass index (BMI) in mothers with HIV (Table 1). Mothers with HIV had medians of 347 CD4^+^ T cells/μL of blood and <20 HIV RNA copies/mL of plasma. All but one of the mothers with HIV reported antiretroviral compliance during pregnancy. There were no differences in alcohol or tobacco use or level of education between the two groups.

**TABLE 1:** Participant characteristics at delivery^*a*^

Characteristic	Value for:		*P*
	HIV or exposed group	Non-HIV or unexposed group	
Mothers	With HIV (*n* = 123)	Without HIV (*n* = 117)	
Age (yrs) (median [Q1, Q3])	30.0 [26.0, 34.5]	25.0 [22.0, 30.0]	<0.01
Previous pregnancies			
Median [Q1, Q3]	2.00 [1.00, 3.00]	1.00 [0, 2.00]	<0.01
No. (%) missing	2 (1.6)	5 (4.3)	
BMI at 62 wks			
Median [Q1, Q3]	23.9 [20.5, 28.1]	27.1 [21.7, 31.1]	0.02
No. (%) missing	51 (41.5)	59 (50.4)	
No. (%) with no smoking during pregnancy	114 (92.7)	113 (96.6)	0.18
No. (%) with no alcohol during pregnancy	112 (91.1)	109 (93.2)	0.55
CD4+ cells/μL			
Median [Q1, Q3]	347 [227, 499]	NA	
No. (%) missing	7 (5.7)	NA	
Log HIV RNA copies/mL			
Median [Q1, Q3]	1.00 [0, 2.06]^*b*^	NA	
No. (%) missing	10 (8.1)	NA	
No. (%) compliant with ART	122 (99.2)	NA	
Infants	HEU (*n* = 123)	HUU (*n* = 117)	
No. (%) female	65 (52.8)	56 (47.9)	0.44
No. (%) with vaginal delivery	116 (94.3)	116 (99.1)	0.07
Gestational age (wks)			
Mean (SD)	39.1 (2.22)	39.3 (1.52)	0.48
No. (%) missing	4 (3.3)	1 (0.9)	
Birth wt (g) [mean (SD)]	3,070 (423)	3,250 (437)	<0.01

a ART, antiretroviral treatment; NA, not evaluated.

b A target that was not detected was assigned a value of 0, and those with values of <20 were assigned a value of 10 copies/mL.

At birth, HEU and HUU had similar gestational ages by study design, with an average of 39 weeks. The sex distribution was also similar (Table 1). HEU had significantly lower weight at birth, with a mean of 3,070 g compared with 3,250 g in HUU. None of the infants were small or large for gestational age. Seven HEU and one HUU were delivered by emergency cesarean section for obstetrical indications identified after initiation of labor.

Feeding pattern and antibiotic usage were recorded at each visit (Table 2). At 6 weeks of life, >80% of infants were exclusively breastfed in each group and 15% HEU and 10% HUU, respectively, were exclusively formula fed. At 28 weeks, 61% of HEU and 50% of HUU were still exclusively breastfed, whereas exclusive formula feeding was reported in 22% of HEU and 11% of HUU. At 62 weeks, all infants received solid foods. Co-trimoxazole was used in 5%, 90%, and 87% of the HEU at 6, 28, and 62 weeks.

**TABLE 2:** Changes in breastfeeding and co-trimoxazole use over time in infants with gut microbiome information

Parameter	No. (%) of infants at:					
	6 wks		28 wks		62 wks	
	HEU (*n* = 98)	HUU (*n* = 88)	HEU (*n* = 86)	HUU (*n* = 80)	HEU (*n* = 74)	HUU (*n* = 74)
Feeding method						
Exclusive breast feeding	83 (84.7)	72 (81.8)	52 (60.5)	40 (50.0)	0 (0)	0 (0)
Exclusive formula feeding	13 (13.3)	4 (4.5)	19 (22.1)	9 (11.3)	0 (0)	0 (0)
Mixed^*a*^	2 (2.0)	12 (13.6)	15 (17.4)	31 (38.8)	74 (100)	74 (100)
Co-trimoxazole						
No	93 (94.9)	88 (100)	8 (9.3)	79 (98.8)	10 (13.5)	74 (100)
Yes	5 (5.1)	0 (0)	77 (89.5)	0 (0)	64 (86.5)	0 (0)
Missing	0 (0)	0 (0)	1 (1.2)	1 (1.3)	0 (0)	0 (0)

a Any combination of breast milk, formula, and/or solids.

### Infant gut microbiota.

**FIG 1:**
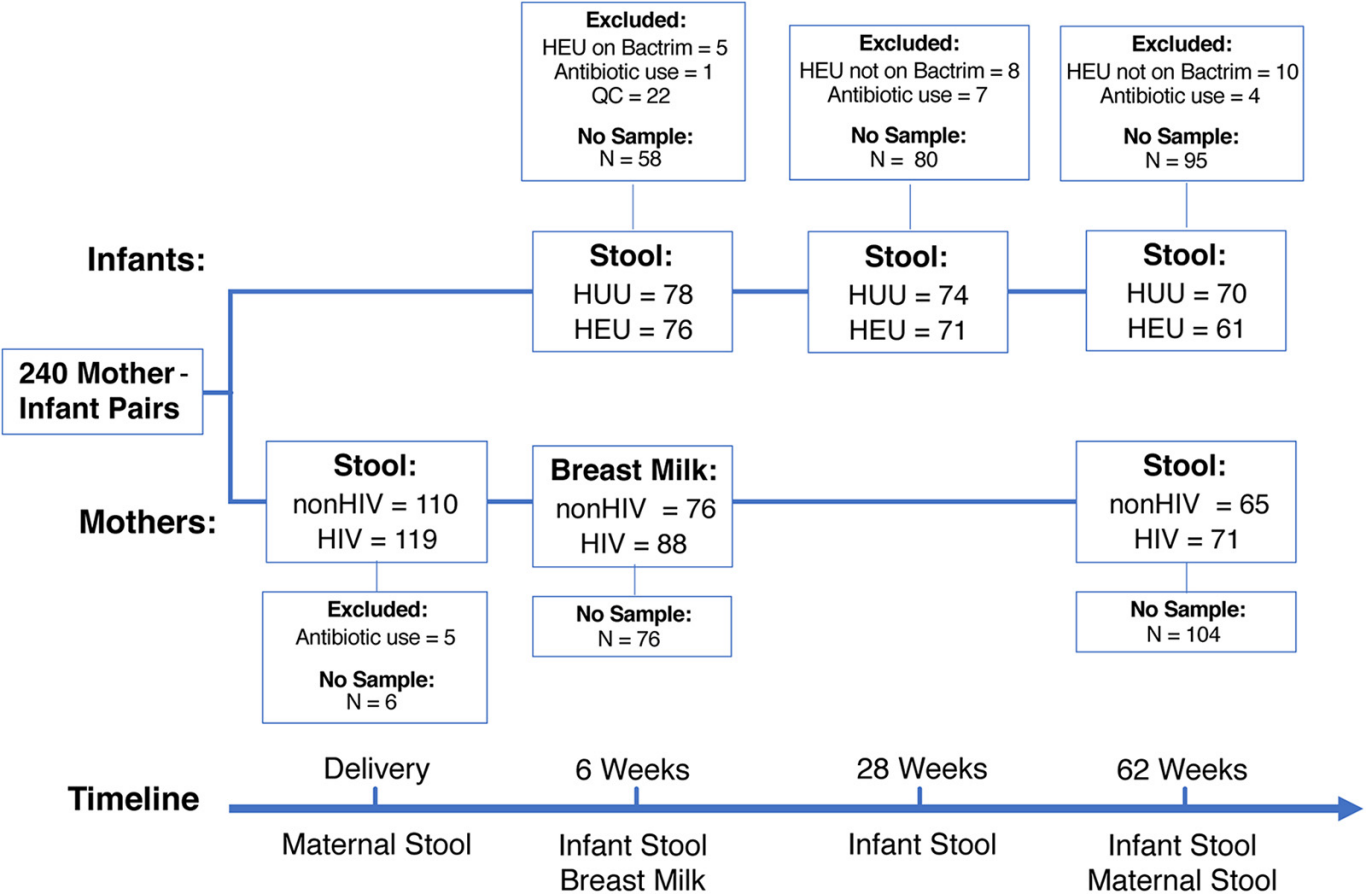
Consort diagram. “QC” indicates samples that were excluded because they did not pass sequence quality control (details are provided in Materials and Methods).

Figure 1 shows the number of samples collected for the microbiome analyses and the reasons for exclusions. Among infants, 61 to 78 rectal samples/group of HEU or HUU were included in the final analysis.

**FIG 2:**
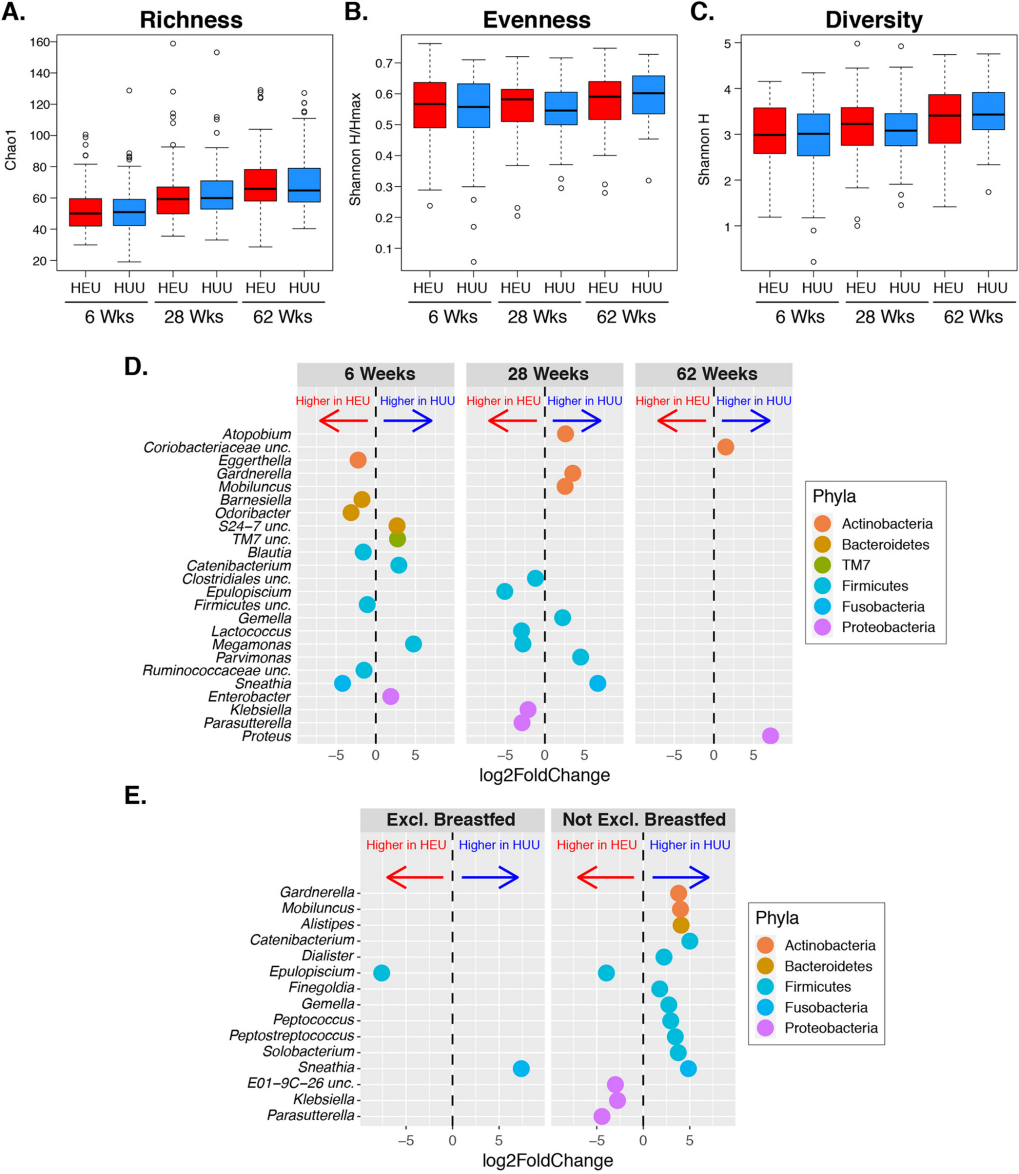
Infant gut microbiome. (A to C) Alpha diversity analysis of community richness (Chao1), evenness (Shannon H/Hmax), and diversity (Shannon H). There were no significant differences between HEU and HUU. (D) Genera with significantly different relative abundances between HEU and HUU at the indicated time points (FDR *P* < 0.05). (E) Genera with significantly different relative abundances between HEU and HUU exclusively (Excl.) breastfed at 28 weeks of life and not exclusively breastfed.

No significant differences were found in either alpha diversity (richness, evenness, and Shannon diversity) or beta diversity between HEU and HUU at any time point (Fig. 2). An evaluation of the relationship between diversity of the HEU gut microbiome at 6 weeks and the maternal CD4^+^ T cell counts or viral loads at delivery did not reveal significant associations (data not shown).

At 6 weeks, 12 out of the 224 genus-level taxa significantly differed between HEU and HUU, including higher abundance in HEU of *Eggerthella sp, Barnesiella sp, Odoribacter sp, Blautia sp, Sneathia sp*, one *Firmicutes uncl*, and one *Ruminococcaceae uncl*, and lower abundance in HEU of *Catenibacterium sp, Megamonas sp, Enterobacter sp*, TM7, and *Bacteroidetes uncl* (Fig. 2). In addition, compared with HUU, the HEU microbiome contained higher overall abundance of *Firmicutes uncl, Carnobacteriaceae* and *Enterococcaceae* families; lower abundance of the *Coriobacteriaceae, Bacteroidetes* and *S24-7* families; and lower abundance of the phylum *Proteobacteria* (Table S1).

At 28 weeks, 12 taxa significantly differed between HEU and HUU. However, the divergent taxa were not conserved between 6 and 28 weeks (Fig. 2). Compared with HUU, HEU had higher abundance of unclassified *Clostridiales*, *Epulopiscium* sp., *Lactococcus* sp., *Megamonas* sp., Klebsiella sp., and *Parasutterella* sp. and lower abundance of *Atopobium* sp., *Gardnerella* sp., *Mobiluncus* sp., *Gemella* sp., *Parvimonas* sp., and *Sneathia* sp. At the family level, the *Leptotrichiaceae* and family XI *incertae sedis* (*Firmicutes*) were significantly less abundant in HEU than HUU (see Table S1 in the supplemental material). There were no differences in abundance at the phylum level. Considering the difference between HEU and HUU in the proportions of exclusively breastfed infants at 28 weeks of life, we performed separate comparisons in exclusively breastfed and nonexclusively breastfed HEU and HUU (Fig. 2). Regardless of breastfeeding status, *Epulopiscium* sp. was significantly more abundant and *Sneathia* sp. was less abundant in HEU than HUU. All other significantly different taxa were present only among nonexclusively breastfed infants. Exclusively breastfed HEU also had a lower abundance of the family *Leptotrichiaceae* than HUU.

10.1128/mbio.01229-22.4 TABLE S1 (A) Differences in infant gut microbiome; (B) differences in maternal gut microbiome; (C) differences in breast milk microbiome. Download Table S1, DOCX file, 0.05 MB. Copyright © 2022 Jackson et al. 2022 Jackson et al. https://creativecommons.org/licenses/by/4.0/ This content is distributed under the terms of the Creative Commons Attribution 4.0 International license.

At 62 weeks of life, 2 taxa differed significantly between the HEU and HUU gut microbiomes, Proteus sp. and unclassified *Coriobacteriaceae*, both with lower abundance in HEU. At the family level, the *Lachnospiraceae* and unclassified *Clostridiales* were more abundant in HEU. There were no differences at the phylum level (Table S1).

The longitudinal analysis of the infants’ microbiome changes over time showed that richness increased significantly from 6 to 28 weeks in HEU (*P* = 0.02) and HUU (*P* = 0.002) (Fig. S1), but after adjustment for breastfeeding status at 28 weeks, the changes lost statistical significance. In contrast, from 28 to 62 weeks, evenness and Shannon diversity increased significantly in HUU (*P* values of 0.005 and 0.003, respectively) but not in HEU (Fig. S1). After adjustment for exclusive-breastfeeding status at 28 weeks, there were no statistically significant differences in either group of infants. In addition to the decrease in the number of significantly different taxa from 12 at 6 weeks and 28 weeks to 2 at 62 weeks, we also observed a decrease in the variability of the HEU versus HUU fold changes obtained from the DESeq analyses from both 6 weeks and 28 weeks relative to 62 weeks. The variance of the log_2_ fold change estimates for all 224 filtered taxa was significantly greater at 6 weeks (*P* value = 0.01) and at 28 weeks (*P* value = 0.02) than at 62 weeks, further indicating more similarity in the gut microbiome between HEU and HUU over time.

10.1128/mbio.01229-22.1 FIG S1 Longitudinal changes in alpha diversity in HEU and HUU gut microbiomes. Richness increased significantly from 6 to 28 weeks in HEU (FDR = 0.02) and HUU (FDR = 0.002); evenness and Shannon diversity increased significantly only in HUU (FDR *P* values of 0.005 and 0.003, respectively). Download FIG S1, PDF file, 0.1 MB. Copyright © 2022 Jackson et al. 2022 Jackson et al. https://creativecommons.org/licenses/by/4.0/ This content is distributed under the terms of the Creative Commons Attribution 4.0 International license.

### Maternal gut microbiome.

**FIG 3:**
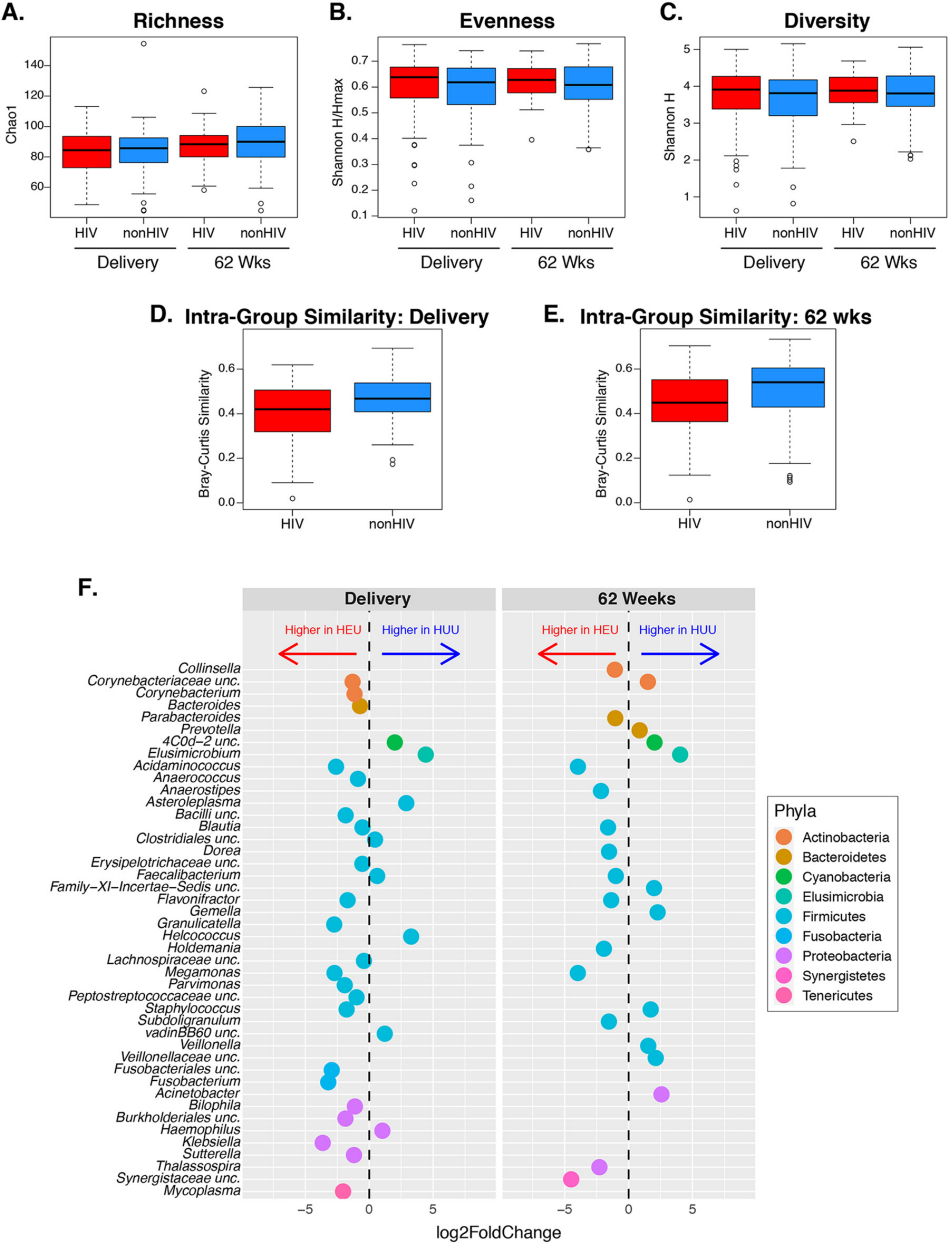
Maternal gut microbiome. (A to C) Alpha diversity analysis of community richness (Chao1), evenness (Shannon H/Hmax), and diversity (Shannon H). There were no significant differences between mothers with and without HIV. (D and E) Beta diversity showing significantly less similarity (higher diversity) among mothers with HIV than among mothers without HIV at delivery (FDR *P* = 0.03) but not postpartum (FDR *P* = 0.06). (F) Genera with significantly different relative abundances between mothers with and without HIV at the indicated time points (FDR *P* < 0.05).

To determine the extent to which the maternal gut microbiome was responsible for differences in the HEU and HUU gut microbiomes, we analyzed the maternal gut microbiota at delivery and 62 weeks postpartum. Alpha diversity of the maternal gut microbiome was similar in mothers with and without HIV at both time points (Fig. 3A to C). However, we found a significant difference in beta diversity between mothers with and without HIV at delivery and 62 weeks postpartum with regard to the microbial composition in the two groups (*P* values of 0.02 and <0.01, respectively) (Fig. S2). To further evaluate the community composition, we assessed interindividual similarity within groups and found that mothers with HIV were less similar to each other when compared to mothers without HIV at either time point, with this difference being statistically significant at delivery (*P* = 0.03) (Fig. 3D) but not at 62 weeks (*P* = 0.06) (Fig. 3E). Finally, the intraindividual similarity for each mother between delivery and postpartum was not different between groups.

10.1128/mbio.01229-22.2 FIG S2 Maternal gut microbiome composition. Bray-Curtis analysis showed significant differences at delivery (FDR = 0.02) and at 62 weeks postpartum (FDR < 0.01). Download FIG S2, PDF file, 0.4 MB. Copyright © 2022 Jackson et al. 2022 Jackson et al. https://creativecommons.org/licenses/by/4.0/ This content is distributed under the terms of the Creative Commons Attribution 4.0 International license.

At delivery, there were 30 taxa significantly different between mothers with and without HIV, 22 of which were significantly more abundant in mothers with HIV (Fig. 3F). Sixteen families differed between mothers with and without HIV, including a higher abundance of *Enterobacteriaceae* and *Carnobacteriaceae* in mothers with HIV, which mirrored the differences between HEU and HUU at 6 weeks (Table S1). Three phyla, *Cyanobacteria*, *Elusimicrobia*, and *Synergistetes*, differed between mothers with and without HIV (Table S1).

At 62 weeks postpartum, 23 taxa significantly differed in abundance between mothers with and without HIV, including 13 with higher abundance in mothers with HIV (Fig. 3F). Consistent differences in microbiome composition between delivery and postpartum included higher abundances of *Blautia* sp., *Megamonas* sp., *Acidaminococcus* sp., and *Flavonifractor* sp. and lower abundance of *Elusimicrobium* sp. in mothers with HIV. Six families and one phylum differed between mothers with and without HIV, all with higher abundance in mothers without HIV (Table S1).

To determine if maternal characteristics, including those associated with HIV infection, played a role in the structure of the maternal gut microbiota and contributed to the differences between mothers with and without HIV, we analyzed the effects of diet, BMI, and age in both groups and of CD4^+^ T cell counts and plasma HIV load at delivery in mothers with HIV. With respect to diet, the analysis of daily consumption of fruit, red meat, any meat, milk, and yogurt showed that mothers with HIV had significantly higher fruit consumption than mothers without HIV at 62 weeks postpartum (averages of 4.66 and 3.92 servings per week) and no other differences (Table S2). We found a significant effect of red meat consumption on overall maternal gut composition (residual permutational multivariate analysis of variance [PERMANOVA] test *P* value < 0.01), but no significant effect of fruit or of any other food group. BMI, which was lower in mothers with HIV (Table 1), also had a significant effect on the overall composition of the maternal gut microbiome at 62 weeks postpartum (residual PERMANOVA test *P* value < 0.01). Although significantly different between mothers with and without HIV, maternal age and parity did not affect the overall composition of maternal gut microbiota. CD4^+^ T cell counts negatively correlated with the intraindividual gut microbiome similarity from delivery to 62 weeks postpartum (*P* = 0.05), but not with the interindividual similarity at delivery (Fig. S3). Plasma HIV load did not have an appreciable effect on the gut microbiome.

10.1128/mbio.01229-22.3 FIG S3 Relationship between the gut microbiome beta diversity and HIV disease characteristics in mothers with HIV. The data show the relationship of intraindividual similarity between delivery and 62 weeks with CD4 count (A) and log HIV load (B) and the relationship between interindividual similarity at delivery and CD4 count (C) and log HIV load (D). Download FIG S3, PDF file, 0.1 MB. Copyright © 2022 Jackson et al. 2022 Jackson et al. https://creativecommons.org/licenses/by/4.0/ This content is distributed under the terms of the Creative Commons Attribution 4.0 International license.

10.1128/mbio.01229-22.5 TABLE S2 Maternal diet was measured as the number of servings per day and then calculated as relative percentage per day. Both counts of the number of servings per day and the relative percentage based on all servings are presented. *P* values are shown for both counts of servings and relative percentage, although only counts were used to determine statistical differences between groups (Wilcoxon test). As these 10 comparisons were planned before the study, they are presented as raw *P* values without adjustment for multiple comparisons. Only fruit consumption at 62 weeks postpartum differed between groups (*P* values = 0.04). Download Table S2, DOCX file, 0.02 MB. Copyright © 2022 Jackson et al. 2022 Jackson et al. https://creativecommons.org/licenses/by/4.0/ This content is distributed under the terms of the Creative Commons Attribution 4.0 International license.

### Maternal breast milk.

**FIG 4:**
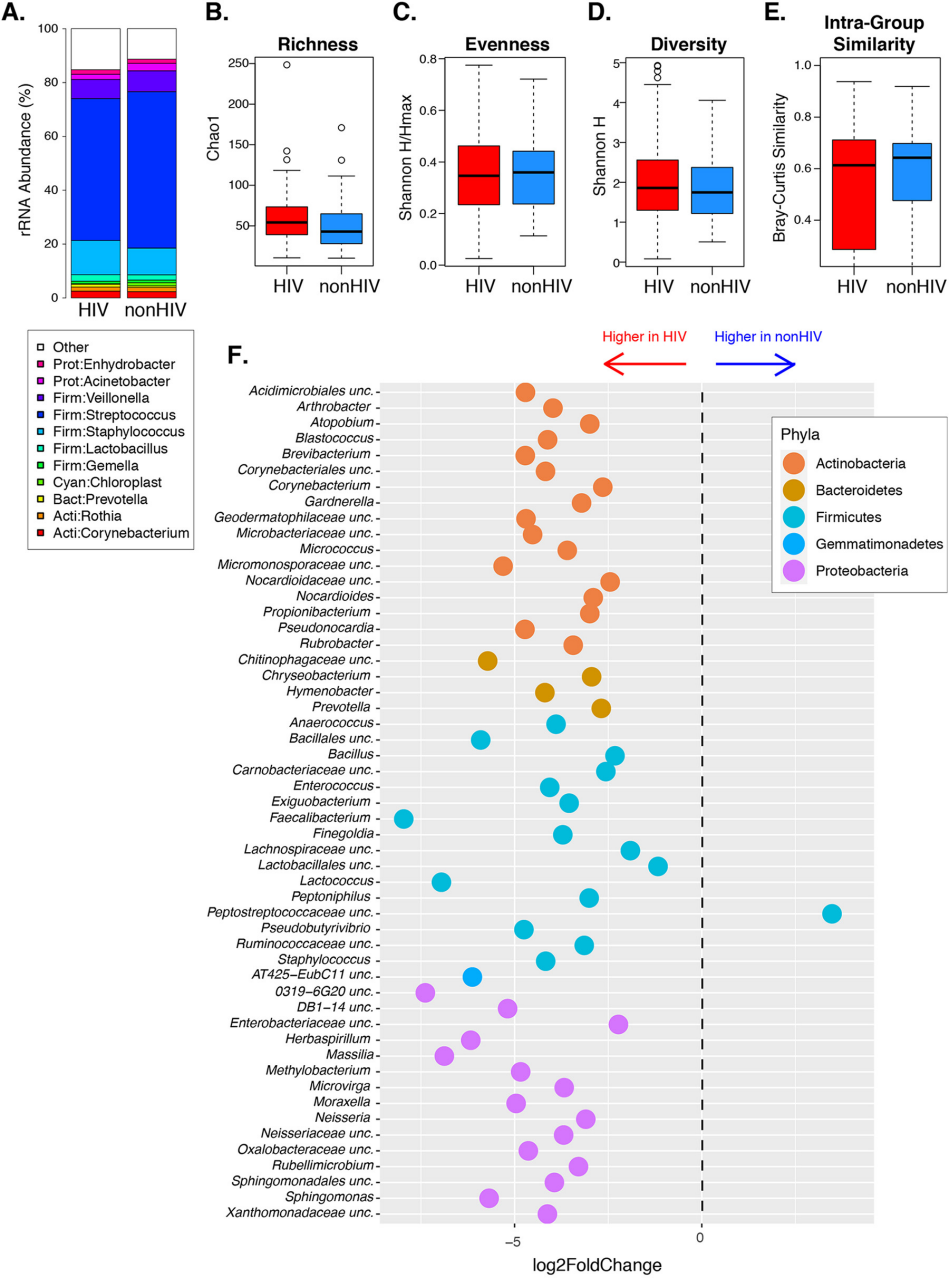
Breast milk microbiome at 6 weeks postpartum. (A) Average relative abundance of bacterial breast milk taxa, stratified by HIV status. (B to D) Alpha diversity analysis of community richness (Chao1), evenness (Shannon H/Hmax), and diversity (Shannon H) showing no significant differences between mothers with and without HIV. (E) Beta diversity showing no differences between mothers with and without HIV. (F) Genera with significantly different abundances in mothers with and without HIV (FDR *P* < 0.05).

Analysis of breast milk-associated bacteria revealed that 50% of the relative abundance across all samples was attributed to Streptococcus or Staphylococcus (mean relative abundance of 55.22% and 11.41%, respectively) (Fig. 4A). The alpha and beta diversity measurements of the breast milk microbiotas were similar in mothers with and without HIV, and there was no difference in interindividual similarity between groups (Fig. 4B to E). Fifty-three taxa were differentially abundant between groups, including 52 with higher abundance in mothers with HIV. Notably, the differentially abundant taxa included the following genera that were also higher in the gut microbiotas of mothers with HIV than without HIV at delivery: Staphylococcus, *Anaerococcus*, and *Corynebacterium*. At the family level, 54 taxa were more abundant in mothers with HIV and none were less abundant compared to mothers without HIV (Table S1). Among the 54 families with differential abundance in maternal breast milk, *Carnobacteriaceae* and *Enterococcaceae* were also more abundant in HEU than HUU gut microbiomes at 6 weeks of life, but the *Coriobacteriaceae* were less abundant in HEU than HUU. There were no differences at the phylum level.

**FIG 5:**
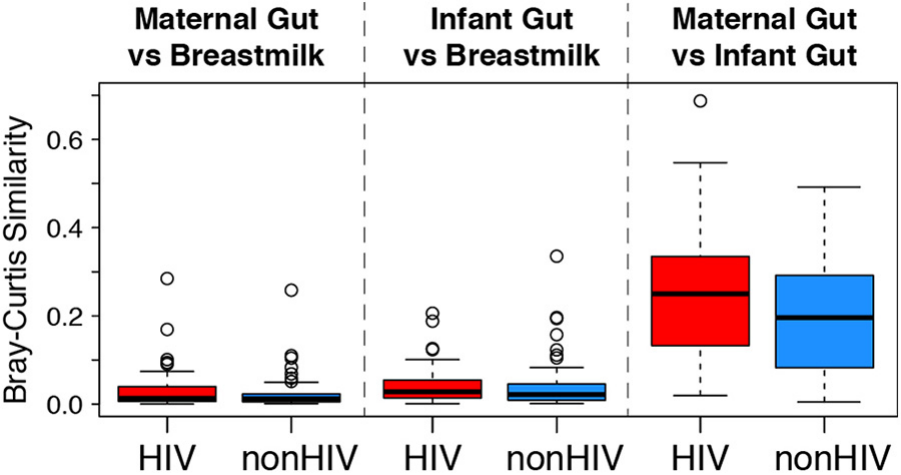
Similarities between breast milk and gut microbiomes, stratified by HIV status. Data were derived from paired analysis of Bray-Curtis similarity scores. The composition of infant gut and breast milk microbiomes at 6 weeks of life had equally little similarity in HEU and HUU mother-infant dyads, which was comparable to the compositions of maternal gut at delivery and breast milk at 6 weeks postpartum. There was equally high similarity between infant and maternal gut microbiomes at 6 weeks of life and delivery, respectively, in HEU and HUU mother-infant dyads.

We performed additional analyses of maternal characteristics that might affect the breast milk microbiome in mothers with and without HIV. Similar to the gut microbiome, red meat consumption was significantly associated with the breast milk microbiome composition. There was no effect of BMI or parity, but there was a significant effect of age on the overall maternal breast milk composition. The analysis of the relationship between the maternal stool and breast milk microbiota composition (intraindividual similarity) did not reveal statistically significant differences between mothers with and without HIV (Fig. 5). There were no statistically significant associations of CD4^+^ T cell count or plasma HIV load at delivery with the breast milk microbiome composition at 6 weeks postpartum.

### Relationship of infant gut microbiota with maternal gut and breast milk microbiota.

At 6 weeks, gut microbiotas in breastfed HEU and HUU were more similar to their respective maternal gut microbiotas sampled at delivery than to maternal breast milk microbiotas (Fig. 5). Moreover, the similarities of the maternal breast milk with both maternal and infant gut microbiotas had low but equal magnitudes.

**FIG 6:**
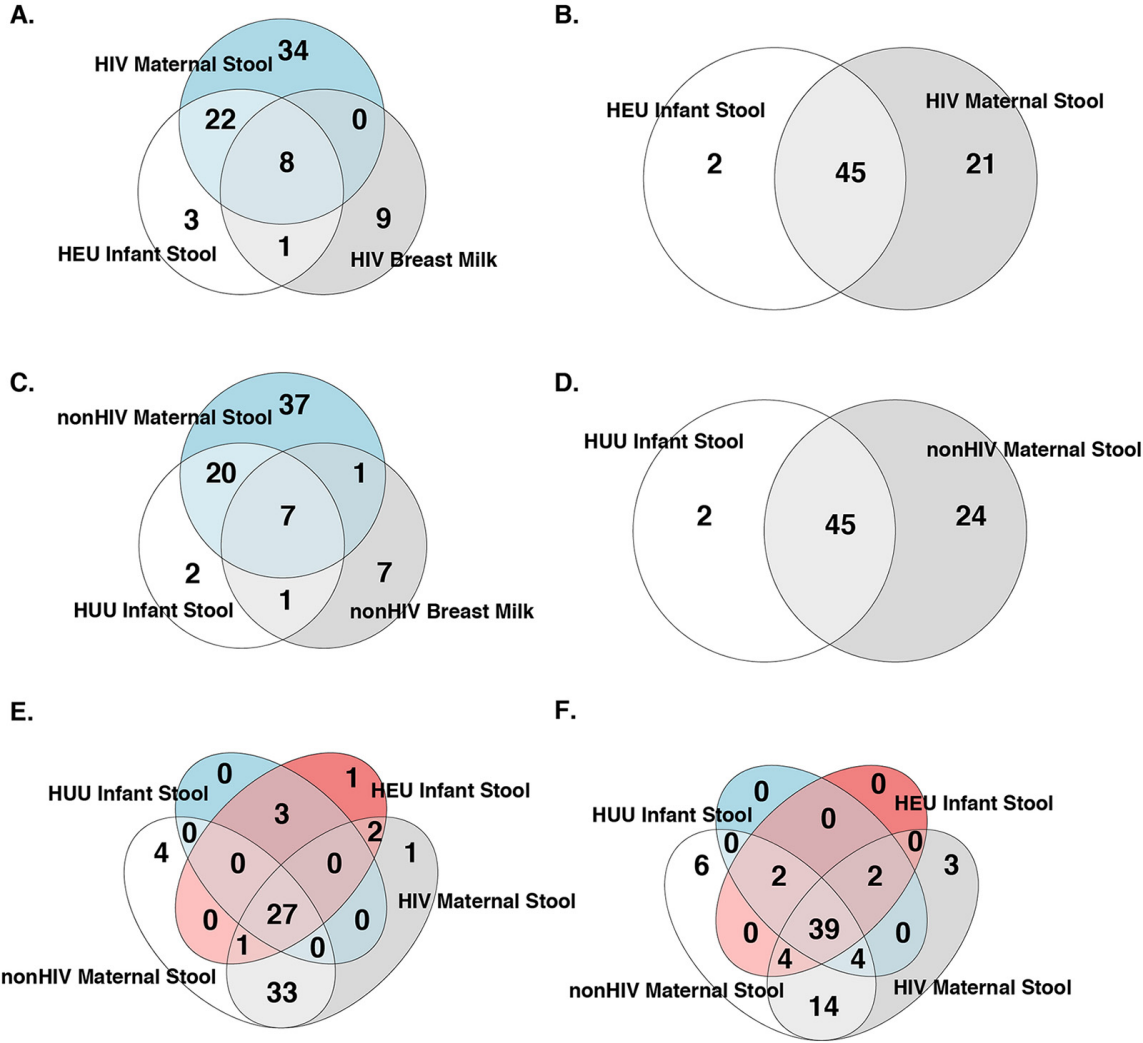
Venn diagrams of core bacterial taxa in infant gut, maternal gut, and breast milk microbiomes. The numerals indicate the numbers of taxa in the shared compartments. (A) HEU gut and breast milk microbiomes at 6 weeks postpartum and maternal gut microbiome at delivery. (B) HEU infant and maternal gut microbiomes at 62 weeks postpartum. (C) HUU gut and breast milk microbiomes at 6 weeks postpartum and maternal gut microbiome at delivery. (D) HUU infant and maternal gut microbiomes at 62 weeks postpartum. (E) HEU and HUU gut microbiomes at 6 weeks of life and maternal gut microbiomes at delivery. (F) HEU, HUU, and maternal gut microbiomes at 62 weeks of life.

At 6 weeks, among 86 core taxa defined by 51% prevalence within a group and ≥0.01% relative abundance, 30 genera were shared between HEU and maternal gut microbiomes, including 8 genera that were also present in the breast milk microbiome. A single genus, *Actinomyces*, was present in HEU gut and breast milk microbiomes and was absent in the maternal gut (Fig. 6A). A similar analysis in HUU revealed 27 genera that were shared between infant and maternal gut microbiomes, including 7 also present in breast milk; the genus that was exclusively shared between HEU gut and breast milk microbiomes, *Actinomyces*, was also exclusively shared between infant gut and breast milk microbiomes in HUU, and a single genus, Haemophilus, was exclusively shared between maternal gut and breast milk microbiomes (Fig. 6C). Twenty-seven core taxa were shared by the gut microbiomes of HEU and HUU infants and their mothers. Two infant gut taxa (*Peptostreptococcus* and *Fusobacterium*) were exclusively shared by HEU and their mothers, but none were exclusively shared by HUU and their mothers (Fig. 6E). In addition, compared with HUU and their mothers, HEU and their mothers at delivery had higher abundances of *Blautia* sp. in the gut microbiome at 6 weeks and Klebsiella sp. and *Megamonas* sp. at 28 weeks. Genera with overlapping differential abundance in breast milk and infant gut microbiome were represented by *Lactococcus* sp. at 28 weeks.

At 62 weeks, 45 taxa were shared between HEU and HUU maternal-infant dyads (Fig. 6B and D). Thirty-nine taxa were shared by the gut microbiomes of HEU with those of HUU and with their mothers (Fig. 6F). No taxa were exclusively shared between HEU or HUU mother-infant gut microbiomes at 62 weeks postpartum.

## DISCUSSION

In this study, which to our knowledge represents the largest analysis of the gut microbiome in HEU infants, we found differences in the relative abundances of multiple individual taxa between HEU and HUU infant gut microbiota, but not in alpha or beta diversity. Our results contrast with a previous report, which showed lower alpha diversity in HEU than HUU gut microbiotas in the first 4 months of life (61). Multiple differences between the two studies may explain the divergent findings, the most notable of which are the following: (i) our study had approximately 5-fold more participants than the previous study (240 versus 48); (ii) our study had a prospective design with inclusion criteria that ensured similar gestational ages at birth and modes of delivery, and homogeneous feeding habits, eliminating many of the confounders that the previous study had to contend with; and (iii) the studies were conducted in different geographic locations (Haiti versus South Africa) (61). We found only two other studies in the literature that compared the gut microbiomes in HEU and HUU. Although each study enrolled only 9 to 25 children/group at around 2 years of age, they found similar alpha and beta diversity in the two groups, supporting our results (63, 64).

As expected, both HEU and HUU groups exhibited significant increases in alpha diversity between 6 and 28 weeks of life. Rapid increases in the diversity of bacterial communities is a well-recognized characteristic of the infant gut microbiome (49, 56, 70). However, in contrast to the HUU gut microbiome, which continued to show increases in evenness and Shannon diversity between 28 and 62 weeks, no significant changes were noted in HEU. This observation was unexpected considering that a higher proportion of HEU than HUU transitioned from exclusive breastfeeding at 28 weeks of life to mixed feeding at 62 weeks. However, all HEU received co-trimoxazole prophylaxis between 28 and 62 weeks, which may have hampered the diversification of the gut microbial communities. A previous study that compared HEU with and without co-trimoxazole prophylaxis showed an increase in richness and prevalence of antibiotic resistance genes associated with the use of co-trimoxazole prophylaxis between 6 and 24 weeks of life, but no information was obtained after 24 weeks (62). Thus, the results of our study could be viewed as complementary to those of the previous study.

Differences in the abundances of specific bacterial taxa between HEU and HUU may be relevant to the immune system development and infectious vulnerability of HEU. For example, Eggerthella lenta, belonging to a genus that was higher in HEU than HUU at 6 weeks of life, has been associated with Th17 cell stimulation, increased risk of rheumatoid arthritis and ulcerative colitis, and enhanced microbial translocation (71, 72). A recent study showed evidence of higher microbial translocation in HEU than HUU in early life, which might be related to the microbiome and, possibly, to the excess of *Eggerthella* sp. in the HEU microbiome in early life (73). Excess abundances of *Carnobacteriaceae* and *Lachnospiraceae*, which were higher in HEU than HUU at 6 and 62 weeks, respectively, were also described in the gut microbiome of adults with HIV versus those without HIV (74). Notably, members of the family *Lachnospiraceae* are producers of butyrate, a short-chain fatty acids that promotes regulatory-T-cell (Treg) differentiation, as are other members of the *Clostridiales*, which were higher in HEU than HUU at 6 weeks of life (34, 75). Increased proportions of Treg may translate into a lower ability to clear infections. Additional research in HEU is needed to better understand relationships between specific gut microbes and the immune responses and infectious disease morbidity in this population.

It is important to note that the abundances of pathobionts, such as Klebsiella sp. and Enterobacter sp., did not differ appreciably between HEU and HUU infants either before or during co-trimoxazole prophylaxis. These findings suggest that gut pathobionts may not contribute to the increased infectious morbidity and mortality observed in HEU.

We found extensive overlap between infant and maternal gut microbiota both in HEU and HUU. However, HEU mother-infant dyads had more taxa in common than HUU dyads at 6 weeks of life, including two taxa, *Peptostreptococcus* sp. and *Fusobacterium* sp., that were exclusively shared by HEU mother-infant pairs. Notably, in this highly treated maternal population with HIV, the maternal CD4^+^ T cell count and HIV plasma RNA at delivery did not influence the maternal or HEU gut microbiota composition. No unique taxa were found in HUU mother-infant dyads that were not present in HEU mother-infant dyads. We found several bacterial taxa with higher abundance in HEU than in HUU maternal-infant dyads, including *Blautia*, Klebsiella, and the family *Lachnospiraceae*, while *Gemella* spp. were less abundant. Other taxa, such as *Clostridiales*, *Megamonas*, and *Parvimonas*, manifested divergent relationships between HEU and HUU compared to mothers with and without HIV. The mechanism underlying the higher similarity between gut microbiota of HEU mother-infant dyads at 6 weeks compared with HUU dyads as well as the biologic relevance of the taxa shared by one group but not the other need to be further investigated, as they may offer clues to HEU-associated immunopathogenesis.

There were more significant differences in the gut microbiome structure between mothers with and without HIV than between HEU and HUU. Significant differences in mothers included higher beta diversity at delivery and 62 weeks postpartum and higher dissimilarity among mothers with HIV than among mothers without HIV. Importantly, although considerable dissimilarity in gut microbiotas were evident between mothers with and without HIV both at delivery and postpartum, the dissimilarity between HEU and HUU gut microbiotas decreased over time. Notably, the excessive morbidity and mortality of infections in HEU also decreases over time, establishing a temporal association with the maturation of the gut microbiome. It will be important to determine in future studies the characteristics of the HEU gut microbiome that are most closely associated with improved protection against infection.

Breast milk plays an important role in shaping the infant gut microbiome. However, at 6 weeks postpartum, we found very little overlap between breast milk and infant gut microbial composition in exclusively breastfed HEU or HUU. In both HEU and HUU, only one genus, *Actinomyces*, was shared between breast milk and infant gut microbiomes but was not also present in the maternal gut microbiome. Actinomyces are common commensals found on mucosae and skin and may have been skin contaminants of breast milk. All other 7 or 8 shared taxa were also present in the maternal gut. These findings suggest that the effect of the breast milk on the infant gut microbiota is mediated by its biochemical and immunologic properties, which favor the development of specific microbial taxa in the infant gut rather than by seeding the infant gut with breast milk bacteria. Although it was proposed that the oligosaccharide composition differed in mothers with and without HIV, exclusive breastfeeding was a unifying factor for the gut microbiota in HEU and HUU, suggesting that differences in oligosaccharide composition may not impact the gut microbiome of HEU versus HUU (76).

Although the primary objective of this study was to compare the HEU and HUU gut microbiomes in the first year of life, the investigation of the maternal gut and milk microbiomes as they related to the infant gut microbiomes revealed notable differences between mothers with and without HIV that are worth emphasizing. The gut microbiomes of mothers with HIV were less similar to one another among members of the group than within groups of mothers without HIV. We hypothesized that mothers with HIV as a group had more diverse immunologic conditions than mothers without HIV, which reduced the constraints normally operating on the gastrointestinal microbiome and thereby expanded person-to-person variation. Since the relationship between the gut microbiome and the host immune system is bidirectional, we thought that immunologic and microbiome dissimilarities within this group might be correlated. We did not find an association between maternal CD4^+^ T cell counts and microbiomes in mothers with HIV. Although this finding does not support our hypothesis, it does not completely refute it either, because the CD4^+^ T cell composition in the intestinal mucosa and/or secondary lymphoid organs may be the key determinant of the gut microbiome, and these parameters cannot be assessed by studying exclusively the peripheral blood. In addition, we did not collect full maternal medical histories, which may have contained important explanatory information, such as occurrence of gastrointestinal infections, nadir CD4^+^ T cell counts in the peripheral blood, or signs of inflammation. The breast milk microbiotas differed between mothers with and without HIV, including an excessive number of taxa with higher abundance in mothers with HIV than in mothers without HIV. Although we did not find any associations of the breast milk microbiome and HIV disease characteristics during the study, differences in immune responses to commensal bacteria between women with HIV and women without HIV might have contributed to the differential abundance of specific taxa. Collectively, these findings indicate that additional studies are needed to fully define the parameters that affect the gut and breast milk microbiomes in mothers with and without HIV.

A limitation of this study was the loss to follow-up among HEU and HUU mother-infant dyads. The use of co-trimoxazole in HEU was an unavoidable confounder. Strengths included the large number of participants enrolled in the study and the excellent quality of the samples that were collected.

We conclude that the gut microbiomes of HEU and HUU are heavily influenced by the maternal gut microbiome, whereas breast milk-associated microbes do not play an apparent, differentiating role. The microbiotas of HEU and HUU converged over time, mirroring the decrease in the excess of infectious morbidity and mortality in HEU. Thus, the immunologic effects of taxa that differentiate the HEU from HUU gut microbiomes in early life deserve further investigation, because to the extent to which they are related to excess infectious morbidity in HEU, they may uncover potential strategies to decrease the vulnerability of HEU to infectious complications in early life.

## MATERIALS AND METHODS

### Study design.

Women with and without HIV were recruited during labor at Chris Hani Baragwanath Hospital in Johannesburg, South Africa. Inclusion criteria for all women were singleton term gestation, planned vaginal delivery, and intent to breastfeed. Women with HIV had to have been prescribed antiretrovirals but not co-trimoxazole during pregnancy. After written informed consent was given, maternal and infant metadata were collected from medical records and by interviewing the study participants at study visits 6, 28, and 62 weeks postpartum. Maternal blood was obtained at delivery for CD4^+^ T cells and HIV plasma RNA measurements at the local laboratory. Infant rectal swabs were obtained at 6, 28, and 62 weeks of life, and maternal rectal swabs were collected at delivery and 62 weeks postpartum for microbiome analysis. Breast milk collected at 6 weeks postpartum from lactating mothers was also submitted to microbiome analysis.

### Microbiome analyses.

DNA was extracted from rectal swabs and breast milk using the QIAamp Powerfecal DNA isolation kit (Qiagen INC, Hilden, Germany). Bacterial profiles were determined by broad-range PCR amplification and sequence analysis of 16S rRNA genes following our previously described methods (77, 78). In brief, amplicons were generated using barcoded primers that target the V3V4 variable region of the 16S rRNA gene: primers 338F (5′-ACTCCTACGGGAGGCAGCAG) and 806R (5′-GGACTACHVGGGTWTCTAAT). PCR products were normalized using a SequalPrep kit (Invitrogen, Carlsbad, CA), pooled, lyophilized, purified, and concentrated using a DNA Clean & Concentrator kit (Zymo, Irvine, CA). Pooled amplicons were quantified using a Qubit 2.0 fluorometer (Invitrogen, Carlsbad, CA). The pool was diluted to 4 nM and denatured with 0.2 N NaOH at room temperature. The denatured DNA was diluted to 15 pM and spiked with 25% Illumina PhiX control DNA prior to loading the sequencer. Illumina paired-end sequencing was performed on the MiSeq platform using a 600-cycle version 3 reagent kit.

Paired-end reads were aligned to human reference genome hg19 with bowtie2, and matching sequences were discarded (79, 80). Demultiplexed paired reads were assembled using Phrap (81, 82) and pairs that did not assemble were discarded. Assembled sequences were trimmed over a moving window of 5 nucleotides until average quality met or exceeded 20. Trimmed sequences with more than one ambiguity or shorter than 350 nucleotides were discarded. Potential chimeras identified with Uchime (usearch6.0.203_i86linux32) (83) using the Schloss (84) Silva reference sequences were removed from subsequent analyses. Assembled sequences were aligned and classified with SINA (1.3.0-r23838) (85) using the 418,497 bacterial sequences in Silva 115NR99 (86) as a reference configured to yield the Silva taxonomy. Taxonomic annotations were based on the default lowest common ancestor parameters used by Silva. Closed-reference operational taxonomic units (OTU) were produced by binning sequences with identical taxonomic assignments. For the gut microbiome, this process generated 89,921,190 sequences for 795 samples (median, 106,528 sequences/sample; interquartile range [IQR], 83,402 to 126,470), and the median Good’s coverage score was ≥99.8%. For the breast milk microbiome, this process generated 11,308,330 sequences for 164 samples (median, 76,650 sequences/sample; IQR, 42,540 to 94,492), and the median Good’s coverage score was ≥99.5%. The software package Explicet (v2.10.5; www.explicet.org) (87) was used to calculate rarefied alpha diversity indices.

### Statistics.

Microbiome analyses were conducted largely within the framework of the microbiome package (version 1.12.0) (88) in R (version 4.0.2) (89), which utilizes both phyloseq (version 1.34.0) (90) and vegan (version 2.5-7) (91). Prior to analysis, the OTU were filtered based on prevalence (at least 5% of libraries are represented) and relative abundance (at least one library must have a relative abundance of 0.01%). Analyses for differences in abundances were conducted using DESeq2 (92), and all significantly different OTU were determined based on a false-discovery-rate (FDR)-adjusted level of 0.05. To determine how the variability of the estimates from DESeq2 changed over time, we followed a multistep procedure: (i) the log_2_ fold change estimates were extracted from each time point for all taxa; (ii) Bartlett’s test was used to test for homogeneity of variances; (iii) an F test was conducted in a pairwise fashion between the three time points if the result of step 2 found a difference in variances. For assessing differences in alpha diversity among two-group comparisons, either Welch’s *t* test or a Wilcoxon test was used as appropriate based on the distribution of the data. Longitudinal alpha diversity measurements were assessed by first calculating the difference in alpha diversity between time points and then fitting a linear model with group as a covariate to determine how each group changed over time. To adjust for breastfeeding, a linear model without a group variable was fit, and then the residuals from that model were used in a regression as the outcome with group as a covariate. A variety of methods were used for the assessment of beta diversity. Overall PERMANOVA were conducted using the Bray-Curtis dissimilarity index and the adonis function (1,000 permutations) within the vegan package (91), followed by pairwise FDR tests if appropriate. To measure both intra- and interindividual similarity, the divergence function within the microbiome package (88) was used with the methodology for beta diversity analyses as described previously (93), with the exception of reporting the similarity instead of dissimilarity by subtracting the calculated divergence from 1.

To assess the global effect of covariates such as diet and BMI on the microbiome, a three-step process was used. First, a logistic regression with group (HIV versus non-HIV) as the outcome and the variable of interest was fitted individually for each variable (red meat, fruit, BMI, etc.), and the residuals were extracted. Next, a matrix of residuals from the DESeq2 analyses for each OTU was extracted. Finally, a PERMANOVA with the adonis function was used with the matrix of the residuals from step 2 on the left side of the equation and the residuals from the model in step 1 on the right side of the equation. The final PERMANOVA yields a *P* value for the global effect of that variable on the microbiome, although further interpretation of this result is not possible from this output alone. Correlations between HIV data and beta diversity used the Spearman rank correlation test to account for the nonparametric nature of the data. Shared core taxa were determined using the eulerr package (version 6.1.1) (94) with the method described on the microbiome package github (88). A prevalence of 51% and a detection limit of 0.0001 were used to match the OTU filtering conducted during the processing of the data. Code for all analyses is available upon request.

### Study approval.

The study was approved by the Human Research Ethics Committee at the University of the Witwatersrand (approval number M171185) and the Colorado Multiple Institutions Review Board. Written informed consent was received prior to participation in the study.

### Data availability.

All sequences and corresponding metadata were deposited in the NCBI Sequence Read Archive under BioProject accession number PRJNA816484.
