# Supplementary material for: Evolution of the Gut Microbiome in HIV-Exposed Uninfected and Unexposed Infants during the First Year of Life
Source: mBio. 2022 Sep 8;13(5):e01229-22. doi: 10.1128/mbio.01229-22 (PMC9600264; doi:10.1128/mbio.01229-22)
Supplement: TABLE S1 [file mbio.01229-22-s0004.docx]

## **Table S1A: Differences in infant gut microbiome**

| **Visit** | **Level** | **Name** | **Log2FoldChange** | **Raw p-value** | **Adjusted p-value** |
| --- | --- | --- | --- | --- | --- |
| 6 Weeks | Genus | Actinobacteria: Eggerthella | -2.224984 | 0.0035860 | 0.0417279 |
|  |  | Bacteroidetes: Barnesiella | -1.741658 | 0.0039520 | 0.0421549 |
|  |  | Bacteroidetes: Odoribacter | -3.116183 | 0.0012425 | 0.0265056 |
|  |  | Bacteroidetes: S24-7 unc. | 2.68156 | 0.0000009 | 0.0000568 |
|  |  | Candidate-division-TM7 unc. | 2.740699 | 0.0002796 | 0.0089468 |
|  |  | Firmicutes unc. | -1.07323 | 0.0016161 | 0.0295513 |
|  |  | Firmicutes: Blautia | -1.580967 | 0.0020054 | 0.0320857 |
|  |  | Firmicutes: Catenibacterium | 2.904708 | 0.0027333 | 0.0377597 |
|  |  | Firmicutes: Megamonas | 4.756287 | 0.0000001 | 0.0000075 |
|  |  | Firmicutes: Ruminococcaceae unc. | -1.481849 | 0.0011809 | 0.0265056 |
|  |  | Fusobacteria: Sneathia | -4.189203 | 0.0002009 | 0.0085737 |
|  |  | Proteobacteria: Enterobacter | 1.888716 | 0.0029500 | 0.0377597 |
|  | Family | Bacteroidetes: S24-7 | 2.497157 | 0.0006143 | 0.0122857 |
|  |  | Actinobacteria: Coriobacteriaceae | 1.391639 | 0.0000004 | 0.0000191 |
|  |  | Firmicutes unc. | -1.913274 | 0.0000004 | 0.0000191 |
|  |  | Firmicutes: Carnobacteriaceae | -3.049915 | 0.0005506 | 0.0122857 |
|  |  | Firmicutes: Enterococcaceae | -2.081633 | 0.0001618 | 0.0053946 |
|  | Phylum | Proteobacteria | 1.08615 | 0.0000527 | 0.0007904 |
| 28  Weeks | Genus | Actinobacteria: Atopobium | 2.601819 | 0.0000000 | 0.0000000 |
|  |  | Actinobacteria: Gardnerella | 3.523838 | 0.0000000 | 0.0000000 |
|  |  | Actinobacteria: Mobiluncus | 2.551025 | 0.0000000 | 0.0000001 |
|  |  | Firmicutes: Clostridiales unc. | -1.184248 | 0.0000021 | 0.0000775 |
|  |  | Firmicutes: Epulopiscium | -5.048908 | 0.0000128 | 0.0003767 |
|  |  | Firmicutes: Gemella | 2.239203 | 0.0000485 | 0.0011873 |
|  |  | Firmicutes: Lactococcus | -2.942823 | 0.0005394 | 0.0113269 |
|  |  | Firmicutes: Megamonas | -2.763565 | 0.0006349 | 0.0116657 |
|  |  | Firmicutes: Parvimonas | 4.510264 | 0.0009282 | 0.0151610 |
|  |  | Fusobacteria: Sneathia | 6.673057 | 0.0015621 | 0.0217347 |
|  |  | Proteobacteria: Klebsiella | -2.119837 | 0.0016264 | 0.0217347 |
|  |  | Proteobacteria: Parasutterella | -2.877825 | 0.0032932 | 0.0403414 |
|  | Family | Firmicutes: Family-XI-Incertae-Sedis | 2.26976 | 0.0003831 | 0.0189639 |
|  |  | Fusobacteria: Leptotrichiaceae | 3.619828 | 0.0000037 | 0.0003627 |
| 62 Weeks | Genus | Actinobacteria: Coriobacteriaceae unc. | 1.494945 | 0.0000693 | 0.0077633 |
|  |  | Proteobacteria: Proteus | 7.138832 | 0.0000002 | 0.0000554 |
|  | Family | Firmicutes: Clostridiales unc. | -1.247256 | 0.0002958 | 0.0147879 |
|  |  | Firmicutes: Lachnospiraceae | -1.065247 | 0.0000146 | 0.0014620 |

Negative values indicate higher abundance in HEUs, positive values indicate

higher abundance in HUUs.

## **Table S1B: Differences in maternal gut microbiome – Delivery**

| **Visit** | **Level** | **Name** | **Log2FoldChange** | **Raw p-value** | **Adjusted p-value** |
| --- | --- | --- | --- | --- | --- |
| Delivery | Genus | Actinobacteria: Corynebacteriaceae unc. | -1.3017879 | 0.0018925 | 0.0127745 |
|  |  | Actinobacteria: Corynebacterium | -1.1503426 | 0.0031510 | 0.0204184 |
|  |  | Bacteroidetes: Bacteroides | -0.7199408 | 0.0061879 | 0.0358017 |
|  |  | Cyanobacteria: 4C0d-2 unc. | 2.0040998 | 0.0004446 | 0.0050557 |
|  |  | Elusimicrobia: Elusimicrobium | 4.4242958 | 0.0000816 | 0.0016527 |
|  |  | Firmicutes: Acidaminococcus | -2.5984368 | 0.0012389 | 0.0101864 |
|  |  | Firmicutes: Anaerococcus | -0.8826283 | 0.0091362 | 0.0493353 |
|  |  | Firmicutes: Asteroleplasma | 2.899007 | 0.0000166 | 0.0006706 |
|  |  | Firmicutes: Bacilli unc. | -1.8460575 | 0.0040353 | 0.0251427 |
|  |  | Firmicutes: Blautia | -0.5293581 | 0.0008040 | 0.0072647 |
|  |  | Firmicutes: Clostridiales unc. | 0.4563384 | 0.0014006 | 0.0101864 |
|  |  | Firmicutes: Erysipelotrichaceae unc. | -0.5403546 | 0.0014462 | 0.0101864 |
|  |  | Firmicutes: Faecalibacterium | 0.6203327 | 0.0003374 | 0.0042039 |
|  |  | Firmicutes: Flavonifractor | -1.6930531 | 0.0000080 | 0.0004314 |
|  |  | Firmicutes: Granulicatella | -2.740093 | 0.0005908 | 0.0059820 |
|  |  | Firmicutes: Helcococcus | 3.281082 | 0.0004681 | 0.0050557 |
|  |  | Firmicutes: Lachnospiraceae unc. | -0.4141235 | 0.0008072 | 0.0072647 |
|  |  | Firmicutes: Megamonas | -2.7191144 | 0.0000308 | 0.0008310 |
|  |  | Firmicutes: Parvimonas | -1.916336 | 0.0002205 | 0.0029772 |
|  |  | Firmicutes: Peptostreptococcaceae unc. | -0.9956908 | 0.0002001 | 0.0029772 |
|  |  | Firmicutes: Staphylococcus | -1.7720825 | 0.0001518 | 0.0027318 |
|  |  | Firmicutes: vadinBB60 unc. | 1.2173932 | 0.0012826 | 0.0101864 |
|  |  | Fusobacteria: Fusobacteriales unc. | -2.9339082 | 0.0000033 | 0.0002713 |
|  |  | Fusobacteria: Fusobacterium | -3.206765 | 0.0000000 | 0.0000000 |
|  |  | Proteobacteria: Bilophila | -1.1238697 | 0.0014205 | 0.0101864 |
|  |  | Proteobacteria: Burkholderiales unc. | -1.8622247 | 0.0000592 | 0.0013711 |
|  |  | Proteobacteria: Haemophilus | 1.0302412 | 0.0082846 | 0.0462795 |
|  |  | Proteobacteria: Klebsiella | -3.6398693 | 0.0000289 | 0.0008310 |
|  |  | Proteobacteria: Sutterella | -1.19499 | 0.0002030 | 0.0029772 |
|  |  | Tenericutes: Mycoplasma | -2.0477612 | 0.0053191 | 0.0319146 |
|  | Family | Actinobacteria: Actinomycetaceae | 1.046425 | 0.0006069 | 0.0059174 |
|  |  | Cyanobacteria: 4C0d-2 unc. | 2.1863705 | 0.0002148 | 0.0027929 |
|  |  | Elusimicrobia: Elusimicrobiaceae | 4.7497077 | 0.0000128 | 0.0003339 |
|  |  | Firmicutes: Aerococcaceae | 1.3075676 | 0.0046457 | 0.0301970 |
|  |  | Firmicutes: Carnobacteriaceae | -1.7406756 | 0.0001229 | 0.0019177 |
|  |  | Firmicutes: Christensenellaceae | 0.8563686 | 0.0020399 | 0.0144648 |
|  |  | Firmicutes: Clostridiales unc. | 0.4601546 | 0.0100263 | 0.0488783 |
|  |  | Firmicutes: Staphylococcaceae | -2.4536963 | 0.0000002 | 0.0000080 |
|  |  | Firmicutes: vadinBB60 | 1.2853906 | 0.0012003 | 0.0104025 |
|  |  | Fusobacteria: Fusobacteriaceae | -2.617072 | 0.0000000 | 0.0000006 |
|  |  | Fusobacteria: Fusobacteriales unc. | -2.4894878 | 0.0000365 | 0.0007119 |
|  |  | Proteobacteria: Enterobacteriaceae | -1.2765847 | 0.0016690 | 0.0130179 |
|  |  | Proteobacteria: Pasteurellaceae | 1.0304571 | 0.0097345 | 0.0488783 |
|  |  | Synergistetes: Synergistaceae | -1.2970827 | 0.0050800 | 0.0304801 |
|  |  | Tenericutes: Mycoplasmataceae | -2.0591148 | 0.0004829 | 0.0053810 |
|  |  | Verrucomicrobia: vadinHA64 unc. | 1.6292961 | 0.0096880 | 0.0488783 |
|  | Phylum | Cyanobacteria: Cyanobacteria | 2.8511272 | 0.0000011 | 0.0000160 |
|  |  | Elusimicrobia: Elusimicrobia | 4.0615856 | 0.0001631 | 0.0012230 |
|  |  | Synergistetes: Synergistetes | -1.4265861 | 0.0017594 | 0.0087971 |

Negative values indicate higher abundance in mothers with HIV, positive values indicate

higher abundance in mothers without HIV.

## **Table S1B continued: Differences in maternal gut microbiome – 62 Weeks**

| **Visit** | **Level** | **Name** | **Log2FoldChange** | **Raw p-value** | **Adjusted p-value** |
| --- | --- | --- | --- | --- | --- |
| 62 Weeks | Genus | Actinobacteria: Collinsella | -1.0735136 | 0.0004108 | 0.0065728 |
|  |  | Actinobacteria: Corynebacteriaceae unc. | 1.5092455 | 0.0001055 | 0.0020638 |
|  |  | Bacteroidetes: Parabacteroides | -1.0409208 | 0.0013616 | 0.0159757 |
|  |  | Bacteroidetes: Prevotella | 0.8656389 | 0.0011882 | 0.0149376 |
|  |  | Cyanobacteria: 4C0d-2 unc. | 2.0292627 | 0.0025377 | 0.0272168 |
|  |  | Elusimicrobia: Elusimicrobium | 4.0318629 | 0.0037240 | 0.0327712 |
|  |  | Firmicutes: Acidaminococcus | -3.9619778 | 0.0000001 | 0.0000097 |
|  |  | Firmicutes: Anaerostipes | -2.1599383 | 0.0000003 | 0.0000141 |
|  |  | Firmicutes: Blautia | -1.6046294 | 0.0000002 | 0.0000097 |
|  |  | Firmicutes: Dorea | -1.5260274 | 0.0000087 | 0.0002552 |
|  |  | Firmicutes: Faecalibacterium | -0.9960555 | 0.0031937 | 0.0312269 |
|  |  | Firmicutes: Family-XI-Incertae-Sedis unc. | 1.993473 | 0.0002812 | 0.0049492 |
|  |  | Firmicutes: Flavonifractor | -1.3626709 | 0.0052200 | 0.0417596 |
|  |  | Firmicutes: Gemella | 2.2715484 | 0.0005013 | 0.0073524 |
|  |  | Firmicutes: Holdemania | -1.928827 | 0.0000482 | 0.0012121 |
|  |  | Firmicutes: Megamonas | -3.969905 | 0.0000001 | 0.0000097 |
|  |  | Firmicutes: Staphylococcus | 1.72829 | 0.0026289 | 0.0272168 |
|  |  | Firmicutes: Subdoligranulum | -1.5376321 | 0.0000553 | 0.0012161 |
|  |  | Firmicutes: Veillonella | 1.538154 | 0.0007847 | 0.0106240 |
|  |  | Firmicutes: Veillonellaceae unc. | 2.1229817 | 0.0000016 | 0.0000571 |
|  |  | Proteobacteria: Acinetobacter | 2.5726287 | 0.0048451 | 0.0406069 |
|  |  | Proteobacteria: Thalassospira | -2.2807535 | 0.0061999 | 0.0474428 |
|  |  | Synergistetes: Synergistaceae unc. | -4.4899504 | 0.0035325 | 0.0327223 |
|  | Family | Actinobacteria: Bifidobacteriaceae | 1.2678914 | 0.0005011 | 0.0119735 |
|  |  | Actinobacteria: Micrococcaceae | 1.5745811 | 0.0002711 | 0.0119735 |
|  |  | Bacteroidetes: Prevotellaceae | 0.9095353 | 0.0008083 | 0.0121250 |
|  |  | Firmicutes: Aerococcaceae | 2.4275628 | 0.0000236 | 0.0021236 |
|  |  | Firmicutes: Family-XI-Incertae-Sedis | 2.2675141 | 0.0005708 | 0.0119735 |
|  |  | Firmicutes: Staphylococcaceae | 1.8326201 | 0.0006652 | 0.0119735 |
|  | Phylum | Bacteroidetes: Bacteroidetes | 0.4021815 | 0.0024334 | 0.0127874 |
|  |  | Candidate-division-TM7 | 1.5477469 | 0.0078333 | 0.0235000 |
|  |  | Cyanobacteria: Cyanobacteria | 1.9402214 | 0.0044842 | 0.0168158 |
|  |  | Elusimicrobia: Elusimicrobia | 4.3748789 | 0.0025575 | 0.0127874 |
|  |  | Synergistetes: Synergistetes | -2.0727651 | 0.0002763 | 0.0041445 |

Negative values indicate higher abundance in mothers with HIV, positive values indicate

higher abundance in mothers without HIV.

**Table S1C: Differences in breastmilk microbiome**

| **Level** | **Name** | **Log2FoldChange** | **Raw p-value** | **Adjusted p-value** |
| --- | --- | --- | --- | --- |
| Genus | Actinobacteria: Acidimicrobiales unc. | -4.710753 | 0.0037228 | 0.0157184 |
|  | Actinobacteria: Arthrobacter | -3.975462 | 0.0000001 | 0.0000015 |
|  | Actinobacteria: Atopobium | -2.992368 | 0.0000661 | 0.0005709 |
|  | Actinobacteria: Blastococcus | -4.119357 | 0.0000005 | 0.0000101 |
|  | Actinobacteria: Brevibacterium | -4.715705 | 0.0000091 | 0.0001147 |
|  | Actinobacteria: Corynebacteriales unc. | -4.175444 | 0.0000116 | 0.0001294 |
|  | Actinobacteria: Corynebacterium | -2.643948 | 0.0000002 | 0.0000041 |
|  | Actinobacteria: Gardnerella | -3.211333 | 0.0003193 | 0.0022472 |
|  | Actinobacteria: Geodermatophilaceae unc. | -4.696236 | 0.0007238 | 0.0044769 |
|  | Actinobacteria: Microbacteriaceae unc. | -4.519998 | 0.0000101 | 0.0001205 |
|  | Actinobacteria: Micrococcus | -3.594476 | 0.0000000 | 0.0000005 |
|  | Actinobacteria: Micromonosporaceae unc. | -5.308455 | 0.0017402 | 0.0082661 |
|  | Actinobacteria: Nocardioidaceae unc. | -2.447648 | 0.0085890 | 0.0326382 |
|  | Actinobacteria: Nocardioides | -2.898785 | 0.0001399 | 0.0011075 |
|  | Actinobacteria: Propionibacterium | -2.99178 | 0.0026891 | 0.0118821 |
|  | Actinobacteria: Pseudonocardia | -4.723969 | 0.0008011 | 0.0044769 |
|  | Actinobacteria: Rubrobacter | -3.434371 | 0.0132390 | 0.0475754 |
|  | Bacteroidetes: Chitinophagaceae unc. | -5.719895 | 0.0014046 | 0.0072130 |
|  | Bacteroidetes: Chryseobacterium | -2.942234 | 0.0011793 | 0.0062240 |
|  | Bacteroidetes: Hymenobacter | -4.191694 | 0.0004861 | 0.0032987 |
|  | Bacteroidetes: Prevotella | -2.684587 | 0.0000040 | 0.0000580 |
|  | Firmicutes: Anaerococcus | -3.893196 | 0.0000000 | 0.0000000 |
|  | Firmicutes: Bacillales unc. | -5.906313 | 0.0000000 | 0.0000000 |
|  | Firmicutes: Bacillus | -2.318132 | 0.0083739 | 0.0324704 |
|  | Firmicutes: Carnobacteriaceae unc. | -2.562148 | 0.0019761 | 0.0091576 |
|  | Firmicutes: Enterococcus | -4.063444 | 0.0000171 | 0.0001708 |
|  | Firmicutes: Exiguobacterium | -3.543975 | 0.0132710 | 0.0475754 |
|  | Firmicutes: Faecalibacterium | -7.961689 | 0.0000000 | 0.0000009 |
|  | Firmicutes: Finegoldia | -3.71256 | 0.0000048 | 0.0000653 |
|  | Firmicutes: Lachnospiraceae unc. | -1.907183 | 0.0026583 | 0.0118821 |
|  | Firmicutes: Lactobacillales unc. | -1.169008 | 0.0073810 | 0.0292166 |
|  | Firmicutes: Lactococcus | -6.955229 | 0.0000009 | 0.0000151 |
|  | Firmicutes: Peptoniphilus | -3.00834 | 0.0001977 | 0.0014449 |
|  | Firmicutes: Peptostreptococcaceae unc. | 3.479616 | 0.0016837 | 0.0082025 |
|  | Firmicutes: Pseudobutyrivibrio | -4.75181 | 0.0006161 | 0.0040366 |
|  | Firmicutes: Ruminococcaceae unc. | -3.139761 | 0.0016695 | 0.0082025 |
|  | Firmicutes: Staphylococcus | -4.171031 | 0.0000000 | 0.0000000 |
|  | Gemmatimonadetes: AT425-EubC11-terrestrial-group unc. | -6.128804 | 0.0001659 | 0.0012605 |
|  | Proteobacteria: 0319-6G20 unc. | -7.385654 | 0.0007870 | 0.0044769 |
|  | Proteobacteria: DB1-14 unc. | -5.187924 | 0.0034414 | 0.0148605 |
|  | Proteobacteria: Enterobacteriaceae unc. | -2.226639 | 0.0007916 | 0.0044769 |
|  | Proteobacteria: Herbaspirillum | -6.172599 | 0.0000248 | 0.0002357 |
|  | Proteobacteria: Massilia | -6.877859 | 0.0000000 | 0.0000000 |
|  | Proteobacteria: Methylobacterium | -4.839673 | 0.0000146 | 0.0001538 |
|  | Proteobacteria: Microvirga | -3.675994 | 0.0123757 | 0.0461056 |
|  | Proteobacteria: Moraxella | -4.96033 | 0.0000600 | 0.0005427 |
|  | Proteobacteria: Neisseria | -3.098293 | 0.0007861 | 0.0044769 |
|  | Proteobacteria: Neisseriaceae unc. | -3.690313 | 0.0000022 | 0.0000344 |
|  | Proteobacteria: Oxalobacteraceae unc. | -4.635206 | 0.0059175 | 0.0239220 |
|  | Proteobacteria: Rubellimicrobium | -3.294494 | 0.0009982 | 0.0054188 |
|  | Proteobacteria: Sphingomonadales unc. | -3.938547 | 0.0001363 | 0.0011075 |
|  | Proteobacteria: Sphingomonas | -5.682569 | 0.0000000 | 0.0000000 |
|  | Proteobacteria: Xanthomonadaceae unc. | -4.124865 | 0.0048039 | 0.0198423 |

Negative values indicate higher abundance in mothers with HIV, positive values indicate

higher abundance in mothers without HIV.

**Table S1C continued: Differences in breastmilk microbiome**

| **Level** | **Name** | **Log2FoldChange** | **Raw p-value** | **Adjusted p-value** |
| --- | --- | --- | --- | --- |
| Family | Acidobacteria: Subgroup-6 unc. | -5.224798 | 0.0174698 | 0.0476449 |
| Family | Actinobacteria: Acidimicrobiales unc. | -4.685416 | 0.0035501 | 0.0133129 |
| Family | Actinobacteria: Coriobacteriaceae | -2.064983 | 0.0010338 | 0.0047712 |
| Family | Actinobacteria: Corynebacteriaceae | -4.347871 | 0.0000000 | 0.0000000 |
| Family | Actinobacteria: Corynebacteriales unc. | -4.153894 | 0.0000122 | 0.0001047 |
| Family | Actinobacteria: Dermabacteraceae | -2.767513 | 0.0045194 | 0.0164344 |
| Family | Actinobacteria: Geodermatophilaceae | -4.944406 | 0.0000000 | 0.0000000 |
| Family | Actinobacteria: Microbacteriaceae | -5.291218 | 0.0000000 | 0.0000000 |
| Family | Actinobacteria: Micromonosporaceae | -6.873277 | 0.0000036 | 0.0000336 |
| Family | Actinobacteria: Propionibacteriaceae | -2.784522 | 0.0033299 | 0.0128899 |
| Family | Actinobacteria: Pseudonocardiaceae | -4.365572 | 0.0007543 | 0.0037716 |
| Family | Actinobacteria: Rubrobacteriaceae | -3.404431 | 0.0122695 | 0.0368085 |
| Family | Bacteroidetes: Cytophagaceae | -3.927412 | 0.0000592 | 0.0004439 |
| Family | Bacteroidetes: Prevotellaceae | -2.031221 | 0.0006164 | 0.0032161 |
| Family | Bacteroidetes: Rikenellaceae | -6.789341 | 0.0026008 | 0.0107620 |
| Family | Firmicutes: Bacillales unc. | -5.189742 | 0.0000000 | 0.0000000 |
| Family | Firmicutes: Carnobacteriaceae | -2.377814 | 0.0000530 | 0.0004244 |
| Family | Firmicutes: Clostridiales unc. | -2.780186 | 0.0128666 | 0.0376582 |
| Family | Firmicutes: Enterococcaceae | -5.049743 | 0.0000000 | 0.0000005 |
| Family | Firmicutes: Family-XI-Incertae-Sedis | -5.009624 | 0.0000000 | 0.0000000 |
| Family | Firmicutes: Family-XII-Incertae-Sedis | -4.137452 | 0.0001174 | 0.0007887 |
| Family | Firmicutes: Lachnospiraceae | -1.892054 | 0.0017764 | 0.0076131 |
| Family | Firmicutes: Lactobacillaceae | -1.58147 | 0.0072289 | 0.0247849 |
| Family | Firmicutes: Lactobacillales unc. | -1.152184 | 0.0082859 | 0.0261660 |
| Family | Firmicutes: Leuconostocaceae | -3.402616 | 0.0113269 | 0.0348520 |
| Family | Firmicutes: Listeriaceae | -5.433699 | 0.0002264 | 0.0012935 |
| Family | Firmicutes: Planococcaceae | -2.331164 | 0.0082129 | 0.0261660 |
| Family | Firmicutes: Ruminococcaceae | -3.084909 | 0.0001615 | 0.0009689 |
| Family | Firmicutes: Staphylococcaceae | -4.191782 | 0.0000000 | 0.0000000 |
| Family | Gemmatimonadetes: AT425-EubC11-terrestrial-group unc. | -6.107088 | 0.0001183 | 0.0007887 |
| Family | Proteobacteria: 0319-6G20 | -7.337263 | 0.0005957 | 0.0032161 |
| Family | Proteobacteria: Acetobacteraceae | -3.783098 | 0.0014868 | 0.0066081 |
| Family | Proteobacteria: Burkholderiaceae | -2.830816 | 0.0074813 | 0.0249378 |
| Family | Proteobacteria: DB1-14 unc. | -5.164905 | 0.0030576 | 0.0122303 |
| Family | Proteobacteria: Enterobacteriaceae | -3.100357 | 0.0000000 | 0.0000002 |
| Family | Proteobacteria: Erythrobacteraceae | -3.470735 | 0.0135024 | 0.0385784 |
| Family | Proteobacteria: Hyphomicrobiaceae | -4.034777 | 0.0150618 | 0.0420330 |
| Family | Proteobacteria: Methylobacteriaceae | -8.26729 | 0.0000000 | 0.0000000 |
| Family | Proteobacteria: Neisseriaceae | -3.082558 | 0.0000021 | 0.0000208 |
| Family | Proteobacteria: Oxalobacteraceae | -6.620798 | 0.0000000 | 0.0000000 |
| Family | Proteobacteria: Pseudomonadaceae | -2.200126 | 0.0010071 | 0.0047712 |
| Family | Proteobacteria: Rhodospirillaceae | -3.51734 | 0.0070100 | 0.0247410 |
| Family | Proteobacteria: Sphingomonadaceae | -5.4735 | 0.0000000 | 0.0000000 |
| Family | Proteobacteria: Sphingomonadales unc. | -3.914302 | 0.0001369 | 0.0008649 |

# Negative values indicate higher abundance in mothers with HIV, positive

values indicate higher abundance in mothers without HIV.
