# Supplementary material for: Evolution of the Gut Microbiome in HIV-Exposed Uninfected and Unexposed Infants during the First Year of Life
Source: mBio. 2022 Sep 8;13(5):e01229-22. doi: 10.1128/mbio.01229-22 (PMC9600264; doi:10.1128/mbio.01229-22)
Supplement: FIG S1 [file mbio.01229-22-s0001.pdf]

Supplemental Figure 1: Longitudinal Alpha Diversity

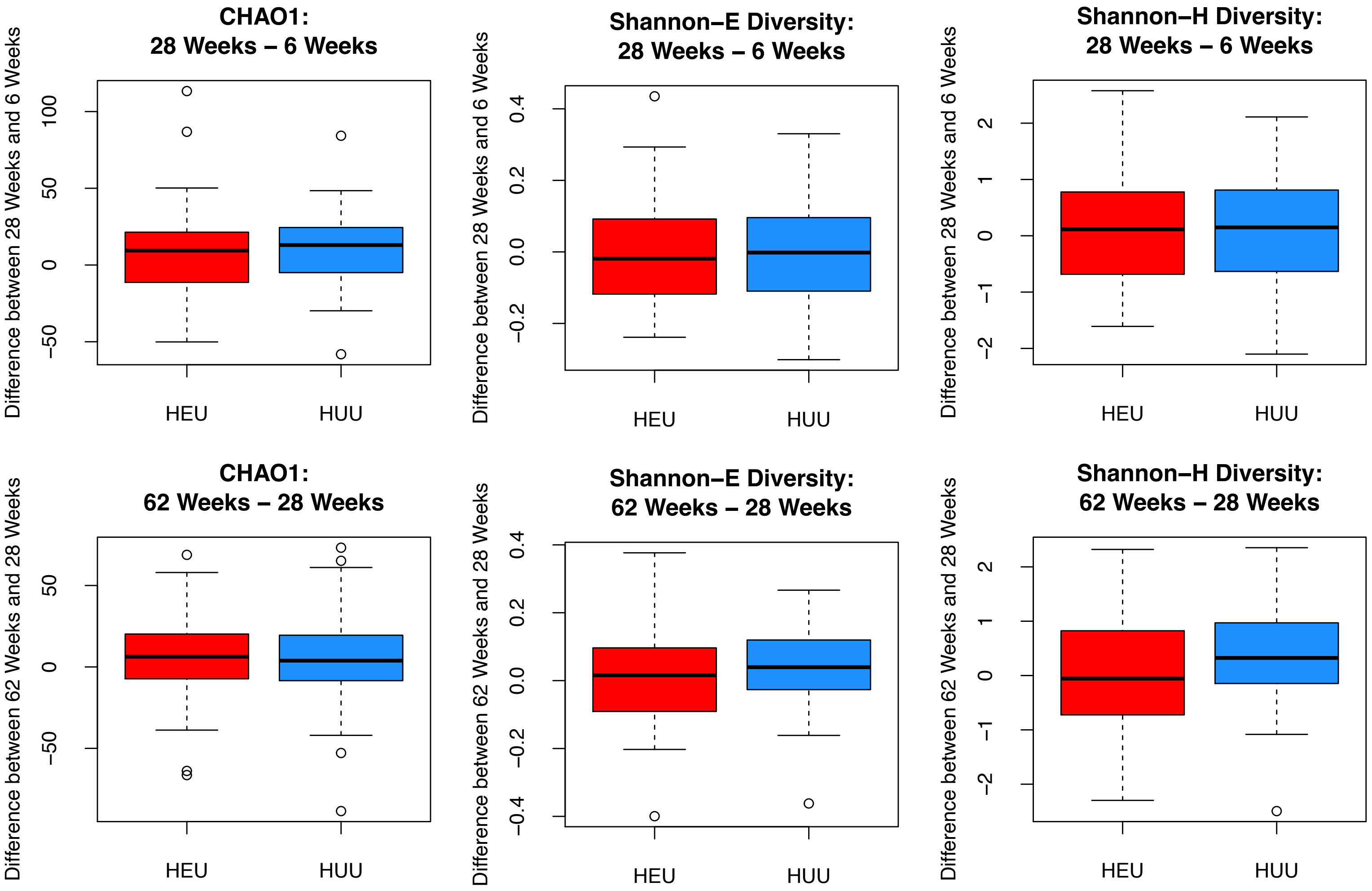

Longitudinal changes in alpha diversity measures between HEUs and HUUS.
