# Supplementary material for: Evolution of the Gut Microbiome in HIV-Exposed Uninfected and Unexposed Infants during the First Year of Life
Source: mBio. 2022 Sep 8;13(5):e01229-22. doi: 10.1128/mbio.01229-22 (PMC9600264; doi:10.1128/mbio.01229-22)
Supplement: FIG S2 [file mbio.01229-22-s0002.pdf]

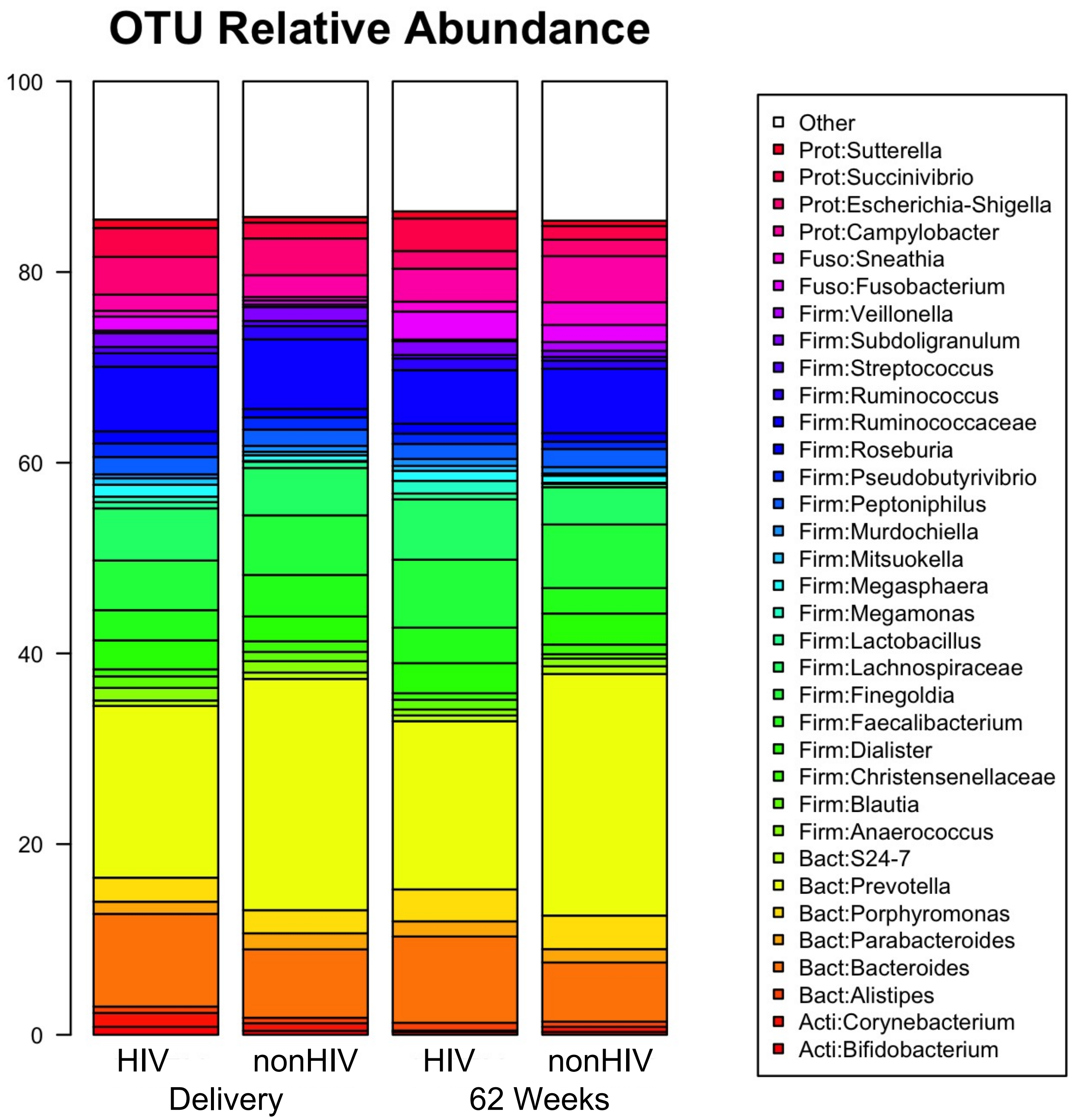

Figure S2: Maternal Gut Microbiome Composition. The Bray-Curtis analysis showed significant differences were observed at delivery (FDR=0.02) and at 62 weeks postpartum (FDR<0.01).
