# Supplementary material for: Evolution of the Gut Microbiome in HIV-Exposed Uninfected and Unexposed Infants during the First Year of Life
Source: mBio. 2022 Sep 8;13(5):e01229-22. doi: 10.1128/mbio.01229-22 (PMC9600264; doi:10.1128/mbio.01229-22)
Supplement: FIG S3 [file mbio.01229-22-s0003.pdf]

Figure S3: Maternal microbiome – Beta diversity and HIV data

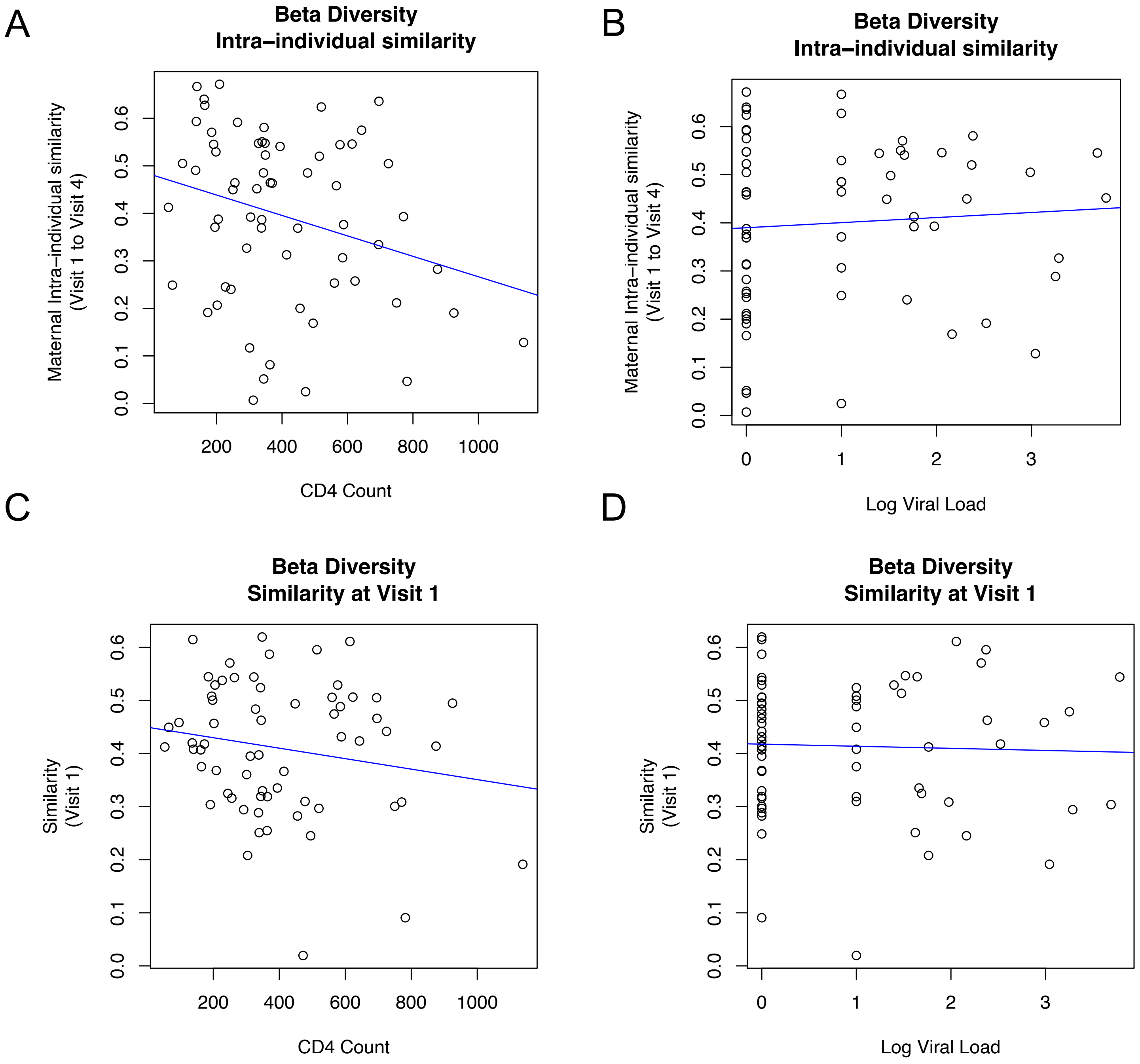

The relationship between paired intra-individual similarity between delivery and 62 weeks and CD4 count (S3A) and log viral load (S3B). The relationship between inter-individual similarity at delivery and CD4 count and log viral load (S3C – D) The relationship between paired intra-individual similarity between delivery and 62 weeks and CD4 count (S3A) and log viral load (S3B). The relationship between inter-individual similarity at delivery and CD4 count and log viral load (S3C – D)
