# Supplementary material for: Evolution of the Gut Microbiome in HIV-Exposed Uninfected and Unexposed Infants during the First Year of Life
Source: mBio. 2022 Sep 8;13(5):e01229-22. doi: 10.1128/mbio.01229-22 (PMC9600264; doi:10.1128/mbio.01229-22)
Supplement: TABLE S2 [file mbio.01229-22-s0005.docx]

## **Table S2: Maternal Diet**

| **Counts** | **Mothers with HIV** | **Mothers without HIV** | **Unadjusted p-value** | **Mothers with HIV** | **Mothers without HIV** | **Unadjusted p-value** |
| --- | --- | --- | --- | --- | --- | --- |
|  | **Delivery** | **Delivery** |  | **62 Weeks** | **62 Weeks** |  |
|  | **(N=119)** | **(N=110)** |  | **(N=71)** | **(N=65)** |  |
| **Fruit** | | | | | | |
| Mean (SD) | 4.43 (1.88) | 4.22 (1.89) | 0.36 | 4.66 (2.09) | 3.92 (2.12) | 0.04 |
| Median [Min, Max] | 5.00 [0, 14.0] | 4.00 [0, 7.00] |  | 4.00 [1.00, 7.00] | 3.00 [1.00, 7.00] |  |
| **Red Meat** | | | | | | |
| Mean (SD) | 1.55 (1.39) | 1.50 (1.54) | 0.56 | 1.32 (1.31) | 1.40 (1.16) | 0.56 |
| Median [Min, Max] | 1.00 [0, 5.00] | 1.00 [0, 7.00] |  | 1.00 [0, 5.00] | 1.00 [0, 5.00] |  |
| **Any Meat** | | | | | | |
| Mean (SD) | 2.96 (1.84) | 2.76 (1.60) | 0.44 | 4.00 (1.98) | 4.45 (1.98) | 0.17 |
| Median [Min, Max] | 3.00 [0, 7.00] | 3.00 [0, 7.00] |  | 4.00 [0, 7.00] | 5.00 [0, 7.00] |  |
| **Milk** | | | | | | |
| Mean (SD) | 3.33 (2.03) | 3.50 (2.32) | 0.73 | 3.73 (2.59) | 3.88 (2.28) | 0.60 |
| Median [Min, Max] | 4.00 [0, 7.00] | 3.00 [0, 7.00] |  | 3.00 [0, 7.00] | 3.00 [0, 7.00] |  |
| **Yogurt** | | | | | | |
| Mean (SD) | 2.29 (1.95) | 2.37 (2.27) | 0.84 | 1.42 (1.98) | 1.40 (1.96) | 0.91 |
| Median [Min, Max] | 2.00 [0, 7.00] | 2.00 [0, 7.00] |  | 1.00 [0, 7.00] | 1.00 [0, 7.00] |  |
| **Relative** | **Mothers with HIV** | **Mothers without HIV** | **Unadjusted p-value** | **Mothers with HIV** | **Mothers without HIV** | **Unadjusted p-value** |
|  | **Delivery** | **Delivery** |  | **62 Weeks** | **62 Weeks** |  |
|  | **(N=119)** | **(N=110)** |  | **(N=71)** | **(N=65)** |  |
| **Fruit** | | | | | | |
| Mean (SD) | 0.314 (0.134) | 0.307 (0.134) | 0.52 | 0.317 (0.145) | 0.267 (0.132) | 0.04 |
| Median [Min, Max] | 0.313 [0, 1.00] | 0.286 [0, 0.778] |  | 0.286 [0.0625, 0.778] | 0.259 [0.0714, 0.778] |  |
| **Red Meat** | | | | | | |
| Mean (SD) | 0.115 (0.109) | 0.114 (0.121) | 0.61 | 0.0870 (0.0818) | 0.0991 (0.0927) | 0.79 |
| Median [Min, Max] | 0.0769 [0, 0.500] | 0.0769 [0, 0.636] |  | 0.0833 [0, 0.333] | 0.0800 [0, 0.400] |  |
| **Any Meat** | | | | | | |
| Mean (SD) | 0.217 (0.143) | 0.207 (0.125) | 0.67 | 0.278 (0.143) | 0.303 (0.129) | 0.26 |
| Median [Min, Max] | 0.200 [0, 1.00] | 0.200 [0, 0.667] |  | 0.278 [0, 0.667] | 0.300 [0, 0.636] |  |
| **Milk** | | | | | | |
| Mean (SD) | 0.211 (0.115) | 0.225 (0.127) | 0.51 | 0.232 (0.146) | 0.252 (0.130) | 0.30 |
| Median [Min, Max] | 0.250 [0, 0.500] | 0.250 [0, 0.538] |  | 0.200 [0, 0.636] | 0.233 [0, 0.778] |  |
| **Yogurt** | | | | | | |
| Mean (SD) | 0.143 (0.105) | 0.147 (0.120) | 0.96 | 0.0853 (0.102) | 0.0794 (0.0866) | 0.99 |
| Median [Min, Max] | 0.125 [0, 0.500] | 0.125 [0, 0.438] |  | 0.0588 [0, 0.438] | 0.0625 [0, 0.350] |  |

Maternal diet was measured as the number of servings per day and then calculated as relative percentage per day. Both counts of the number of servings per day and the relative percentage based on all servings are presented. P-values are shown for both counts of servings and relative percentage, although only counts were used to determine statistical differences between groups (Wilcoxon test). As these 10 comparisons were planned before data collection, they are presented as raw p-values without adjustment for multiple comparisons. Only fruit consumption at 62 weeks postpartum differed between groups (p-value = 0.04).
